# Promoter regulatory mode evolution enhances the high multidrug resistance of *tmexCD1-toprJ1*

**DOI:** 10.1128/mbio.00218-24

**Published:** 2024-04-02

**Authors:** Chengzhen Wang, Jun Yang, Zeling Xu, Luchao Lv, Sheng Chen, Mei Hong, Jian-Hua Liu

**Affiliations:** 1 State Key Laboratory for Animal Disease Control and Prevention, Guangdong Laboratory for Lingnan Modern Agriculture, College of Veterinary Medicine, South China Agricultural University, Guangzhou, China; 2 Key Laboratory of Zoonosis of Ministry of Agricultural and Rural Affairs, National Risk Assessment Laboratory for Antimicrobial Resistant of Microorganisms in Animals, Guangdong Provincial Key Laboratory of Veterinary Pharmaceutics Development and Safety Evaluation, Guangzhou, Guangdong, China; 3 Guangdong Province Key Laboratory of Microbial Signals and Disease Control, Integrative Microbiology Research Centre, South China Agricultural University, Guangzhou, China; 4 State Key Lab of Chemical Biology and Drug Discovery and the Department of Food Science and Nutrition, The Hong Kong Polytechnic University, Kowloon, Hong Kong, China; 5 College of Life Sciences, South China Agricultural University, Guangzhou, China; 6 Guangdong Provincial Key Laboratory of Protein Function and Regulation in Agricultural Organisms, South China Agricultural University, Guangzhou, China; Louis Stokes Veterans Affairs Medical Center, Cleveland, Ohio, USA

**Keywords:** antibiotic resistance, tigecycline, gene regulation, evolution, *tmexCD-toprJ*

## Abstract

Antibiotic resistance could rapidly emerge from acquiring the mobile antibiotic resistance genes, which are commonly evolved from an intrinsic gene. The emergence of the plasmid-borne mobilized efflux pump gene cluster *tmexCD1-toprJ1* renders the last-resort antibiotic tigecycline ineffective, although its evolutionary mechanism remains unclear. In this study, we investigate the regulatory mechanisms of the progenitor NfxB-MexCD-OprJ, a chromosomally encoded operon that does not mediate antibiotic resistance in the wild-type version, and its homologs, TNfxB1-TMexCD1-TOprJ1 mediating high-level tigecycline resistance, and TNfxB3-TMexCD3-TOprJ1. Mechanistic studies demonstrated that in *nfxB-mexCD-oprJ*, MexCD expression was under a weaker promoter, P*mexC* and inhibited by a strong repressor NfxB. For *tmexCD1-toprJ1*, TMexCD1 was highly expressed owing to the presence of a strong promoter, P*tmexC1*, and an inactive suppressor, TNfxB1, with a T39R mutation that rendered it unable to bind to promoter DNA. In *tnfxB3-tmexCD3-toprJ1b*, TMexCD3 expression was intermediate because of the local regulator TNfxB3, which binds to two inverted repeat sequences of P*tmexC*. Additionally, TNfxB3 exhibited lower protein expression and weaker DNA binding affinity than its ancestor NfxB, together with their promoter activities difference explaining the different expression levels of *tmexCD-toprJ* homologs. Distinct fitness burdens on these homologs-carrying bacteria were observed due to the corresponding expression level, which might be associated with their global prevalence. In summary, our data depict the mechanisms underlying the evolution and dissemination of an important mobile antibiotic resistance gene from an intrinsic chromosomal gene.

**IMPORTANCE:**

As antibiotic resistance seriously challenges global health, tigecycline is one of the few effective drugs in the pipeline against infections caused by multidrug-resistant pathogens. Our previous work identified a novel tigecycline resistance efflux pump gene cluster *tmexCD1-toprJ1* in animals and humans, together with its various variants, a rising clinical concern. Herein, this study focused on how the local regulation modes of *tmexCD1-toprJ1* evolved to a highly expressed efflux pump. Through comparative analysis between three *tnfxB-tmexCD-toprJ* homologs and their progenitor *nfxB-mexCD-oprJ*, modes, we demonstrated the evolutionary dynamics from a chromosomal silent gene to an active state. We found the de-repression of the local regulator and an increase of the promoter activity work together to promote a high production of drug efflux machines and enhance multidrug resistance. Our findings revealed that TMexCD1-TOprJ1 adopts a distinct evolutionary path to achieve higher multidrug resistance, urgently needing tight surveillance.

## INTRODUCTION

Bacterial antimicrobial resistance (AMR) is a growing threat to public health, and few effective drugs are currently available to treat complex bacterial infections (1). This threat is exacerbated by the widespread presence of antibiotic resistance genes (ARGs) in various biomes (2). Increasing anthropogenic activities, including clinical and animal-fed drug usage (3), exacerbate the continuous emergence and dissemination of ARGs, particularly those mediating multidrug resistance (MDR). Some ARGs influence the intracellular drug concentrations by decreasing influx and increasing efflux (4). The active ejection of antibiotics facilitated by efflux pump systems decreases the intracellular drug concentration and is regarded as a crucial molecular mechanism of AMR (5, 6). Substantial chromosome-encoded efflux pump machines have been widely identified in clinical pathogens. These include tripartite (resistance–nodulation–division) efflux pump systems, such as AcrAB-TolC in Enterobacteriaceae and MexAB-OprM, MexCD-OprJ in *Pseudomonas* (7). These pump systems not only involve AMR but also contribute to the diverse physiological functions of bacteria (8). However, the expression of these efflux systems and the expulsion of drugs are energy-dependent and generally modulated by complex regulatory networks (6).

Tigecycline is the first glycylcycline-class antimicrobial to be used as a last-resort treatment for counteracting multidrug-resistant bacterial infections (9). Since the start of the clinical use of tigecycline, two main resistance mechanisms have emerged: tetracycline-inactivating enzymes and the overexpression of chromosomally encoded efflux pumps (10–12). In 2020, we reported a novel plasmid-borne efflux pump gene cluster, *tmexCD1-toprJ1*, which confers tigecycline resistance in *Klebsiella* spp. (13). Subsequently, various *tmexCD-toprJ* homologs have been identified in *Klebsiella* spp., *Aeromonas* spp., *Proteus* spp., and *Pseudomonas* spp. (14–16). In addition to tigecycline resistance, *tmexCD-toprJ* gene clusters can confer resistance to tetracyclines, cephalosporins, aminoglycosides, and quinolones. Currently, *tmexCD-toprJ* gene clusters, especially *tmexCD1-toprJ1*, *tmexCD2-toprJ2*, and *tmexCD3-toprJ1b*, have been identified in a diverse range of clinically important bacterial species in humans, animals, and the environment, posing a threat to public health (17–19).

**Fig 1:**
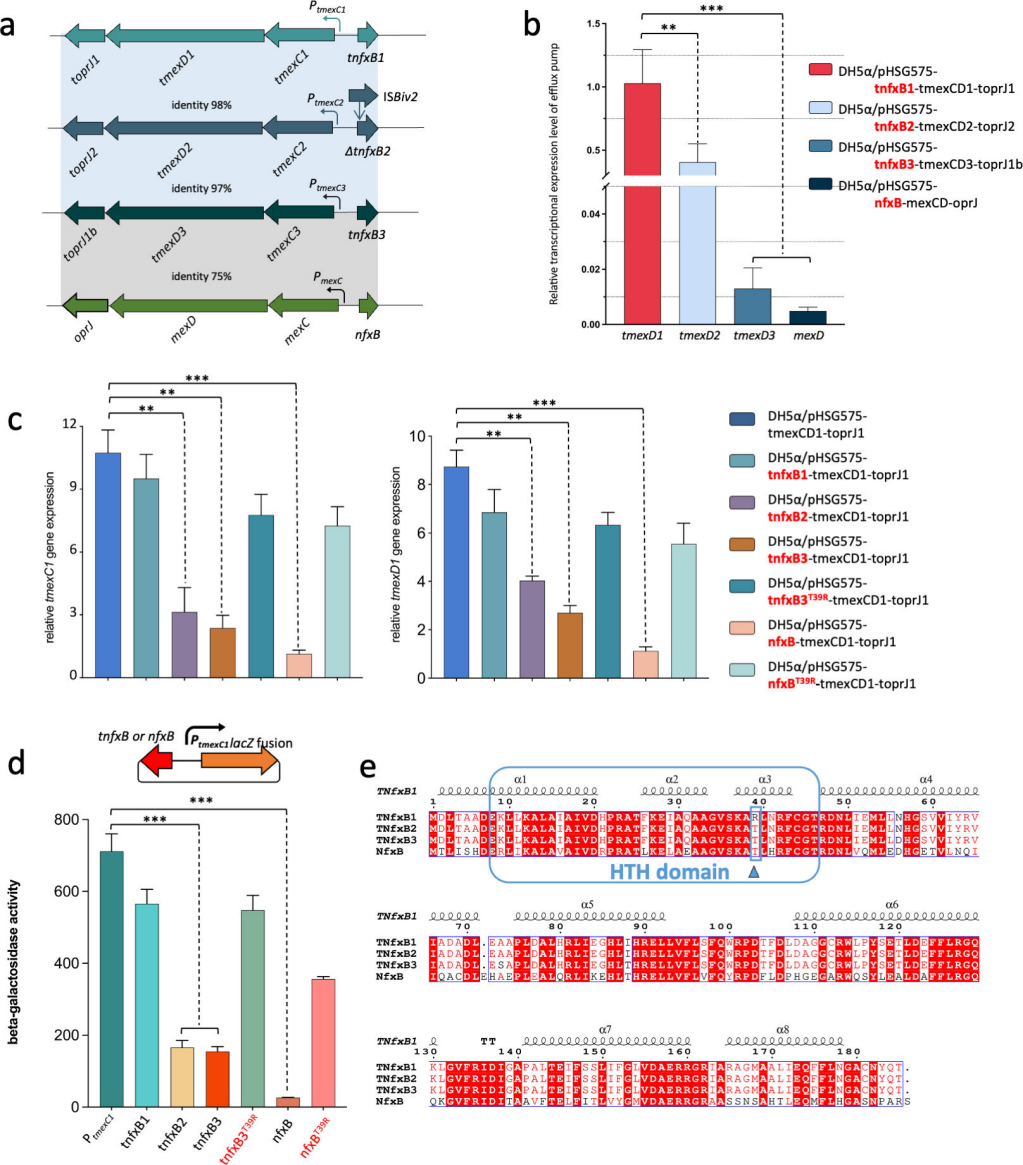
Characterization of the regulation by TNfxB and NfxB. (**a**) Linear comparison of *tnfxB-tmexCD-topJ* operons and *mexCD-oprJ* operon. The nucleotide sequence identity was indicated with colored shadows. (**b**) Relative transcriptional expression level of efflux pump gene in four recombinant strains carrying different gene operons. The radio pf expression level was determined by RT-qPCR and compared with strain DH5α/pHSG575-tnfxB1-tmexCD1-toprJ1. 16S rRNA gene was used to normalize the gene expression. (**c**) The effect of TNfxB or NfxB on the relative mRNA expression of genes *tmexC1* and *tmexD1* measured by RT-qPCR. The transcriptional expression level was normalized by 16S rRNA gene and the fold changes were obtained relative to that of DH5α/pHSG575-nfxB-tmexCD1-toprJ1. (**d**) *In vivo* β-galactosidase assay of the repression effects of TNfxB, NfxB, and its mutant on the expression of *lacZ* fusion with the promoter of *tmexC1*. (**e**) Alignment of amino acid sequence encoded by three TNfxB proteins with NfxB from *P. aeruginosa* PAO1. All results were presented as mean ± SD and the significances were measured by unpaired *t* test. *, *P*  <  0.05; **, *P*  <  0.01; and ***, *P*  <  0.001.

*tmexCD-toprJ* shows a close (75% nucleotide identity) evolutionary relationship to *mexCD-oprJ* in *Pseudomonas* and is speculated to originate from this inherent efflux system (13). Similar to the chromosomal *nfxB-mexCD-oprJ* operon, *tmexCD-toprJ* genes are generally accompanied by a potential regulator, *tnfxB* (20). NfxB negatively controls the expression of the *mexCD-oprJ* efflux system (21), resulting in a quiescent state of *mexCD-oprJ*. Our previous studies showed that TNfxB1 did not change the drug resistance phenotype of *tmexCD1-toprJ1*, and in *tnfxB2-mexCD2-toprJ2*, the open reading frame of TNfxB2 was incomplete due to the insertion of IS*Biv2* (Fig. 1a) (13, 14), indicating that the regulation patterns of *tnfxB-tmexCD-toprJ* might be different from those of *nfxB-mexCD-oprJ*. Herein, we compare the functional properties of three TNfxB homologs and NfxB on their targeted transporter expression profiles and illustrate that the transition of *tmexCD-toprJ* regulation modes leads to the gradual evolution of antibiotic resistance, which will provide clues for strategies to combat bacterial infections.

## RESULTS

### Diverse MDR levels and expression profiles of the *tmexCD-toprJ* and *mexCD-oprJ* operons caused by differences in upstream regulators

Based on our previous studies, three novel MDR transporter gene clusters, *tmexCD1-toprJ1*, *tmexCD2-toprJ2*, and *tmexCD3-toprJ1b* (Fig. 1a), display different antibiotic resistance phenotypes. To further compare the antimicrobial resistance phenotypes mediated by these efflux pumps, we cloned the three *tmexCD-toprJ* operons, as well as the intrinsic *mexCD-oprJ* of *Pseudomonas aeruginosa*, together with their corresponding regulators (TNfxB*/*NfxB) into the low-copy-number vector pHSG575 to mimic the wild-type plasmid. These plasmids were transformed into *Escherichia coli* DH5α, and antibiotics susceptibility testing results revealed that the strain carrying *tnfxB1-tmexCD1-toprJ1* possessed the highest minimum inhibitory concentration (MIC) levels for all the tested antibiotics, followed by *tnfxB2-tmexCD2-toprJ2* and *tnfxB3-tmexCD3-toprJ1b* (Table 1). No MIC increases against any drugs were observed for *nfxB-mexCD-oprJ* compared with the control group (Table 1).

**TABLE 1:** Various antibiotic susceptibility profiles (MICs, mg/L) of strains in this study

Strains	Strain information	TIG^*a*^	TET	CQM	FEP	STR
DH5α	Recipient strain	0.25	0.5	0.03	0.015	1
DH5α/pHSG575	Recombinant strain with empty vector	0.25	0.5	0.03	0.015	1
DH5α/pHSG575- tnfxB1-tmexCD1-toprJ1	Transformants expressing *tnfxB1-tmexC1D1-toprJ1* operon with an intergenic sequence of *tnfxB1-tmexC1*	4	4	1	0.5	16
DH5α/pHSG575- tnfxB2-tmexCD2-toprJ2	Transformants expressing *tnfxB2-tmexC2D2-toprJ2* operon with an intergenic sequence of *tnfxB2-tmexC2*	2	2	0.5	0.25	8
DH5α/pHSG575- tnfxB3-tmexCD3-toprJ1b	Transformants expressing *tnfxB3-tmexC3D3-toprJ1b* operon with an intergenic sequence of *tnfxB3-tmexC3*	1	2	0.25	0.125	4
DH5α/pHSG575- nfxB-mexCD-oprJ	Transformants expressing *nfxB-mexCD-toprJ* operon with an intergenic sequence of *nfxB-mexC*	0.25	0.5	0.03	0.015	1
DH5α/pHSG575- tmexCD1-toprJ1	Transformants expressing *tmexC1D1-toprJ1* in promoter of *tmexC1*	4	4	1	0.5	16
DH5α/pHSG575- tnfxB1-tmexCD1-toprJ1	Transformants expressing *tnfxB1-tmexC1D1-toprJ1* operon in promoter of *tmexC1*	4	4	1	0.5	16
DH5α/pHSG575- tnfxB2-tmexCD1-toprJ1	Transformants expressing *tnfxB2-tmexC1D1-toprJ1* operon in promoter of *tmexC1*	2	2	0.5	0.25	8
DH5α/pHSG575- tnfxB3-tmexCD1-toprJ1	Transformants expressing *tnfxB3-tmexC1D1-toprJ1* operon in promoter of *tmexC1*	2	2	0.5	0.25	8
DH5α/pHSG575- tnfxB3^T39R^-tmexCD1-toprJ1	Transformants expressing *tnfxB3 ^T39R^-tmexC1D1-toprJ1* operon in promoter of *tmexC1*	4	4	1	0.5	8
DH5α/pHSG575- nfxB-tmexCD1-toprJ1	Transformants expressing *nfxB-tmexC1D1-toprJ1* operon in promoter of *tmexC1*	0.5	1	0.125	0.125	2
DH5α/pHSG575- nfxB^T39R^-tmexCD1-toprJ1	Transformants expressing *nfxB ^T39R^-tmexC1D1-toprJ1* operon in promoter of *tmexC1*	4	4	1	0.25	8

^*a*^ TIG, tigecycline; TET, tetracycline; CQM, cefquinome; FEP, cefepime; STR, streptomycin.

Given that efflux pump-mediated antibiotic resistance is highly correlated with the expression of efflux pump genes, we next selectively measured the expression of *mexD* and its homologs, *tmexD1*, *tmexD2*, and *tmexD3* using RT-qPCR. As shown in Fig. 1b, the gene displaying the highest expression level was *tmexD1* in the strain carrying *tnfxB1-tmexCD1-toprJ1*, which was followed by *tmexD2* in the strain carrying *tnfxB2-tmexCD2-toprJ2*. Substantially lower efflux pump mRNA levels were found in *tnfxB3-tmexCD3-toprJ1b* and *nfxB-mexCD-oprJ* (Fig. 1b). These results were consistent with the observed MIC levels, indicating a correlation between high transporter expression and high MIC levels (Table 1). However, in the absence of the upstream genes *tnfxB* or *nfxB*, the transcriptional expression of *tmexD1* in the *tmexCD1-toprJ1* operon was similar to that of *tmexD2* in *tmexCD2-toprJ2* (Fig. S1). Moreover, *mexD* in *mexCD-oprJ* exhibited a fivefold lower expression level than *tmexD1* in *tmexCD1-toprJ1*. In the presence of an upstream regulator, the expression of *tmexD1* in *tnfxB1-tmexCD1-toprJ1* was over 100-fold higher than that of *mexD* in the *nfxB-mexCD-oprJ* operon (Fig. 1b; Fig. S1). These results illustrate that the diverse mRNA levels of efflux pumps are strongly dependent on the local regulatory effects of *tnfxB*/*nfxB*.

Next, we investigated the regulatory effect of the three TNfxB proteins and NfxB on the expression of *tmexCD1-toprJ1* by constructing recombinant plasmids, pHSG575-tnfxB-tmexCD1-toprJ1 and pHSG575-nfxB-tmexCD1-toprJ1. The results revealed only slight decreases in the transcriptional expression of *tmexC1* and *tmexD1* in the presence of *tnfxB1*, whereas the transcript levels of *tmexCD1* showed a two- to fivefold decrease in the presence of TNfxB2 or TNfxB3 (Fig. 1c). In contrast, the mRNA levels of *tmexC1* and *tmexD1* were significantly downregulated (9- to 11-fold) by NfxB (Fig. 1c). Moreover, similar effects of NfxB and TNfxB on the activity of the promoter P*_tmexC1_* were observed in a β-galactosidase (β-gal) assay. NfxB could strongly reduce the activity of P*_tmexC1_* by approximately 20-fold, while approximately 4-fold decreases of the promoter activity were observed in the presence of TNfxB2 or TNfxB3 (Fig. 1d). However, TNfxB1 only caused a slight decrease (Fig. 1d). These results are consistent with the antibiotics susceptibility testing results, in which TNfxB2 or TNfxB3 resulted in a twofold decrease in MIC for various antibiotics relative to their MIC levels in the absence of TNfxB, whereas no MIC changes were observed in strains bearing *nfxB-tmexCD1-toprJ1* (Table 1). Taken together, our results illustrate that NfxB represses the expression of *tmexCD1-toprJ1* more strongly than the repressors TNfxB3 and TNfxB2, whereas TNfxB1 shows only slight repressive function.

### Threonine 39 is essential for TNfxB and NfxB function

To compare the functions of these regulators, we aligned three TNfxB isoforms (TNfxB1, TNfxB2, and TNfxB3) with NfxB (Fig. 1e). One different residue was identified between TNfxB1 [arginine 39 (R39)] and TNfxB2 [threonine 39 (T39)], while four amino acids (residues 39, 56, 73, and 88) differed between TNfxB1 and TNfxB3 (Fig. 1e). T39 was also identified in TNfxB3 and NfxB (Fig. 1e). Further bioinformatics analysis revealed that both TNfxB and NfxB belong to the TetR family of regulators (TFRs), which contain an N-terminal helix-turn-helix (HTH) DNA-binding domain, where the R39 and T39 residues are located (Fig. 1e). To determine the effects of these four amino acids, we induced site-directed mutations in TNfxB1 (Table S1). Comparison of the MICs of these four TNfxB1 mutants revealed that only the arginine-to-threonine mutation in residue 39 led to a decrease in antimicrobial MICs in TNfxB1 (Table S1). The four residues in TNfxB3 were also individually mutated to the corresponding residues in TNfxB1; the threonine-to-arginine substitution in TNfxB3 residue 39 (T39R) led to a twofold increase in MIC, but the other three TNfxB3 mutants caused no phenotypic changes (Table S1). The RT-qPCR and β-gal activity results also showed that only the mutations in residue 39 of TNfxB1 or TNfxB3 led to changes in regulation effects (Fig. S2; Fig. 1c). Therefore, T39 is critical for the functions of TNfxB2 and TNfxB3. Because T39 is also conserved in NfxB (Fig. 1e), it was mutated to arginine (NfxB^T39R^) to assess the role of T39 in the function of NfxB. The RT-qPCR and β-gal activity results confirmed that T39 is also critical for the repression function of NfxB (Table 1; Fig. 1c and d).

**Fig 2:**
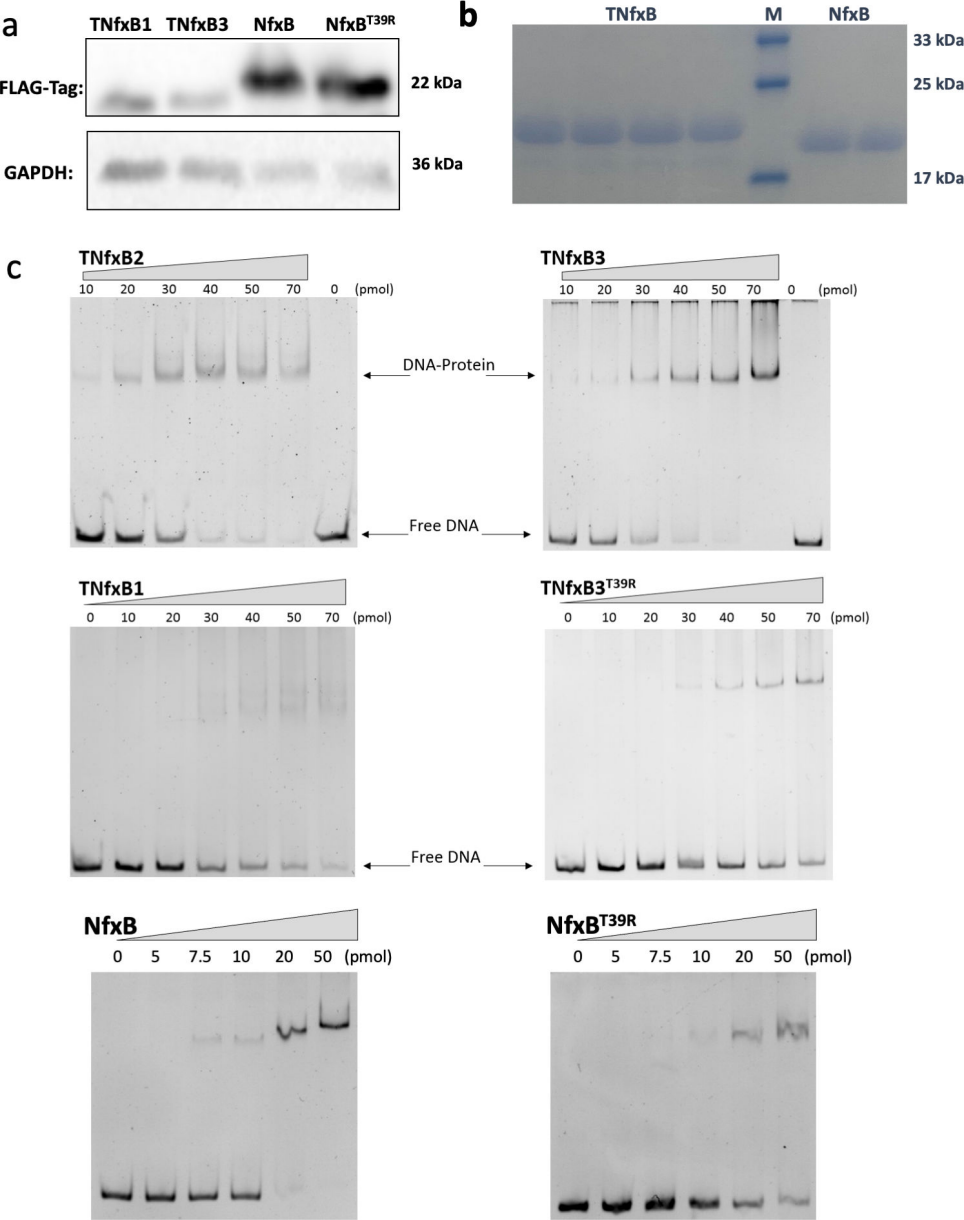
Interaction between TNfxB or NfxB protein and the operator DNA. (**a**) Western blot analysis of FLAG-tagged TNfxB and NfxB protein expression levels in *E. coli* recombinants. GDDPH was used as a control. (**b**) SDS-PAGE analysis of purified four His-tagged TNfxB proteins (TNfxB1, TNfxB2, TNfxB3, and TNfxB3^T39R^), NfxB and its mutant (NfxB^T39R^). M indicated protein marker with the molecular weight shown on the right side. (**c**) Electrophoretic mobility shift assay of His-tagged TNfxB and NfxB protein with the 200 bp DNA fragment spanning the *tnfxB1-tmexC1* intergenic region. The protein concentration of lanes was shown. The DNA and DNA-protein complex were visualized by staining with ethidium bromide.

In addition, to assess whether this residue substitution affected protein expression, TNfxB or NfxB in pHSG575-tnfxB/nfxB-tmexCD1-toprJ1 was tagged with FLAG at the C-terminal end. The FLAG tag had no impact on regulator function (Table S2). In the western blot assay, TNfxB1 and TNfxB3 displayed similar protein levels, as did NfxB and NfxB^T39R^ (Fig. 2a), indicating that T39R substitution does not affect protein expression. Moreover, the protein levels of TNfxB3 and TNfxB1 were markedly lower than those of NfxB and NfxB^T39R^ (Fig. 2a), although they all displayed similar mRNA expression levels (Fig. S3). These results suggest that the protein expression of TNfxB and NfxB differs, which may also contribute to the diverse regulatory effects on efflux pump expression.

### T39R substitution weakens DNA interaction *in vitro*

Because TFR functions by binding to its target operator DNA, we purified four TNfxB proteins, TNfxB1, TNfxB2, TNfxB3, and TNfxB3^T39R^ (Fig. 2b), to examine protein-DNA interactions. Electrophoretic mobility shift assay (EMSA) was conducted using purified TNfxB proteins and a 200-bp intergenic sequence between *tnfxB1* and *tmexC1*. The DNA fragment was gradually upshifted by TNfxB3 owing to the formation of the DNA–protein complex (Fig. 2c). Upshift was not observed using a negative control probe (Fig. S4), suggesting that TNfxB3 binds specifically to P*_tmexC1_*. In contrast, other proteins with R39, TNfxB1, and TNfxB3^T39R^, impaired DNA–protein interaction, whereas TNfxB2, with T39, bound to the promoter of *tmexC1*with a similar binding affinity as TNfxB3 (Fig. 2c). NfxB and NfxB^T39R^ were also purified to investigate their DNA-binding affinities (Fig. 2b). As shown in Fig. 2c, the amount of free DNA decreased gradually with the addition of NfxB, whereas NfxB^T39R^ showed weaker DNA-binding ability, resulting in a smaller band shift than the wild-type NfxB (Fig. 2c). This indicates that the T39R substitution impaired the affinity between NfxB and the operator DNA. These results reveal that T39 is critical for the DNA-binding of TNfxB and NfxB and that the R39 mutation greatly affects DNA–TNfxB and DNA–NfxB affinity, consistent with the MIC and β-gal results.

To determine the location of T39 in the TNfxB3 and NfxB proteins, we searched for resolved TFR structures that showed homology with TNfxB3, and only LfrR in *Mycolicibacterium smegmatis* (Protein Data Bank number: 2V57) was found. This conserved threonine was also found in the LfrR HTH motif (Fig. S5 and S6). Further protein modeling revealed that TNfxB3 superimposed on NfxB displayed a root mean square deviation (RMSD) of 1.457, whereas an RMSD of 0.825 was achieved in the HTH domains of TNfxB3 and NfxB (Fig. S7). Intriguingly, a hydrogen bond was formed between the hydroxyl group of T39 in NfxB and its operator DNA (Fig. S8). To further confirm the critical role of T39, three other point mutants, NfxB^T39D^, NfxB^T39A^, and NfxB^T39S^, were constructed to measure the effects of these mutations with different side chains on NfxB function. We found the serine substitution (T39S) caused little change relative to the wild-type P*_mexC_-nfxB lacZ* fusion, while the NfxB mutant with 39 alanine generated higher β-gal activity (Fig. S9). Additionally, the mutation of residue 39 to aspartate promoted a level similar to that observed in the absence of NfxB (Fig. S9). These results suggest that threonine or serine with a short-chain hydroxyl group is crucial for the DNA-binding function of NfxB.

**Fig 3:**
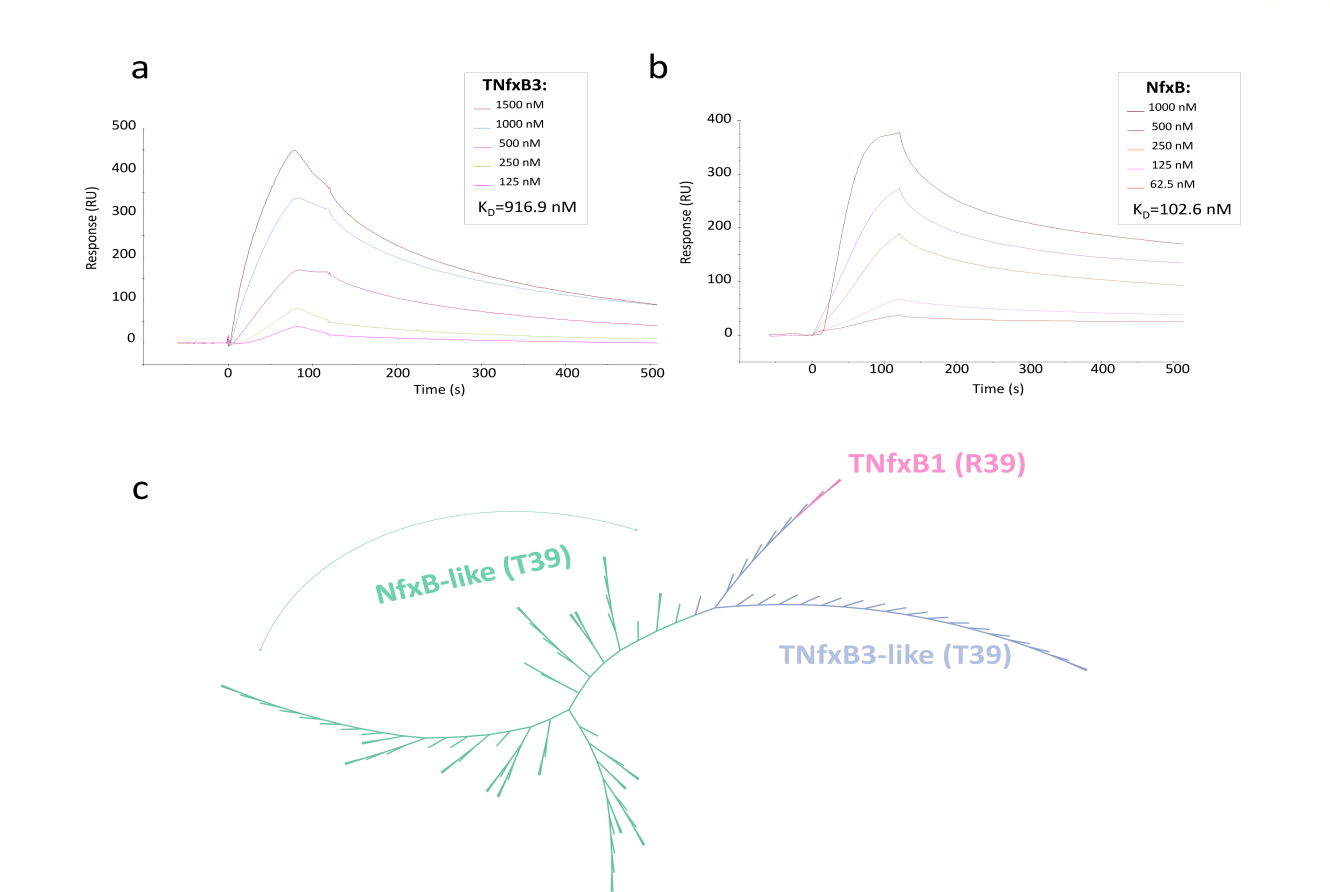
DNA-protein binding analysis of TNfxB3 and NfxB and their phylogenetic relationship. (**a, b**) Surface plasmon resonance analyses of TNfxB3 and NfxB binding to the inverted repeat sequences. (**c**) An unrooted radial phylogenic tree of 95 TNfxB and NfxB homologs. A conserved residue 39 was shown in brackets.

Importantly, we found that 20 pmol of NfxB was sufficient to completely shift the promoter DNA fragment, whereas 50 pmol of TNfxB2 and TNfxB3 were required (Fig. 2c), suggesting that TNfxB binds much less efficiently than NfxB. Next, we complemented this approach using surface plasmon resonance assays. The binding affinity between TNfxB3 and promoter DNA (*K*_*D*_ = 916.9 nM) was 8.9-fold lower than that of NfxB with an active interaction (*K*_*D*_ = 102.6 nM) (Fig. 3a and b). These data demonstrate that NfxB exhibits stronger operator DNA-binding activity than TNfxB3.

### Residue 39 threonine is highly conserved across NfxB and TNfxB homologs, except for the T39R variation in TNfxB1

To examine the conservation of position 39 among TNfxB-like homologs, a broader set of 95 TNfxB-like homologs with greater than 60% amino acid identity was selected to construct a phylogenetic tree (Fig. 3c). Among these, 28 TNfxB-like proteins with 95% amino acid identity were identified in diverse bacterial species, whereas the remaining 67 NfxB-like family proteins were all from *Pseudomonas* spp. The phylogenetic groups showed that the TNfxB homologs could be attributed to the same phylogenetic subgroups as the NfxB-like homologs, indicating that TNfxB proteins originated and evolved from NfxB. A common feature of NfxB and the TNfxB3-like proteins is the presence of T39, whereas TNfxB1 has an R39 residue (Fig. 3c). Sixteen TNfxB-like proteins were used for further analysis. The majority of the TNfxB2- and TNfxB3-like homologs were from *Pseudomonas* spp. and were present immediately upstream of *tmexCD-toprJ*. In contrast, the TNfxB1 homologs were from Enterobacteriaceae, with *Klebsiella* spp. as the main host species (Fig. S10).

### Defining the region within the P*_tmexC1_* element that interacts with TNfxB3

**Fig 4:**
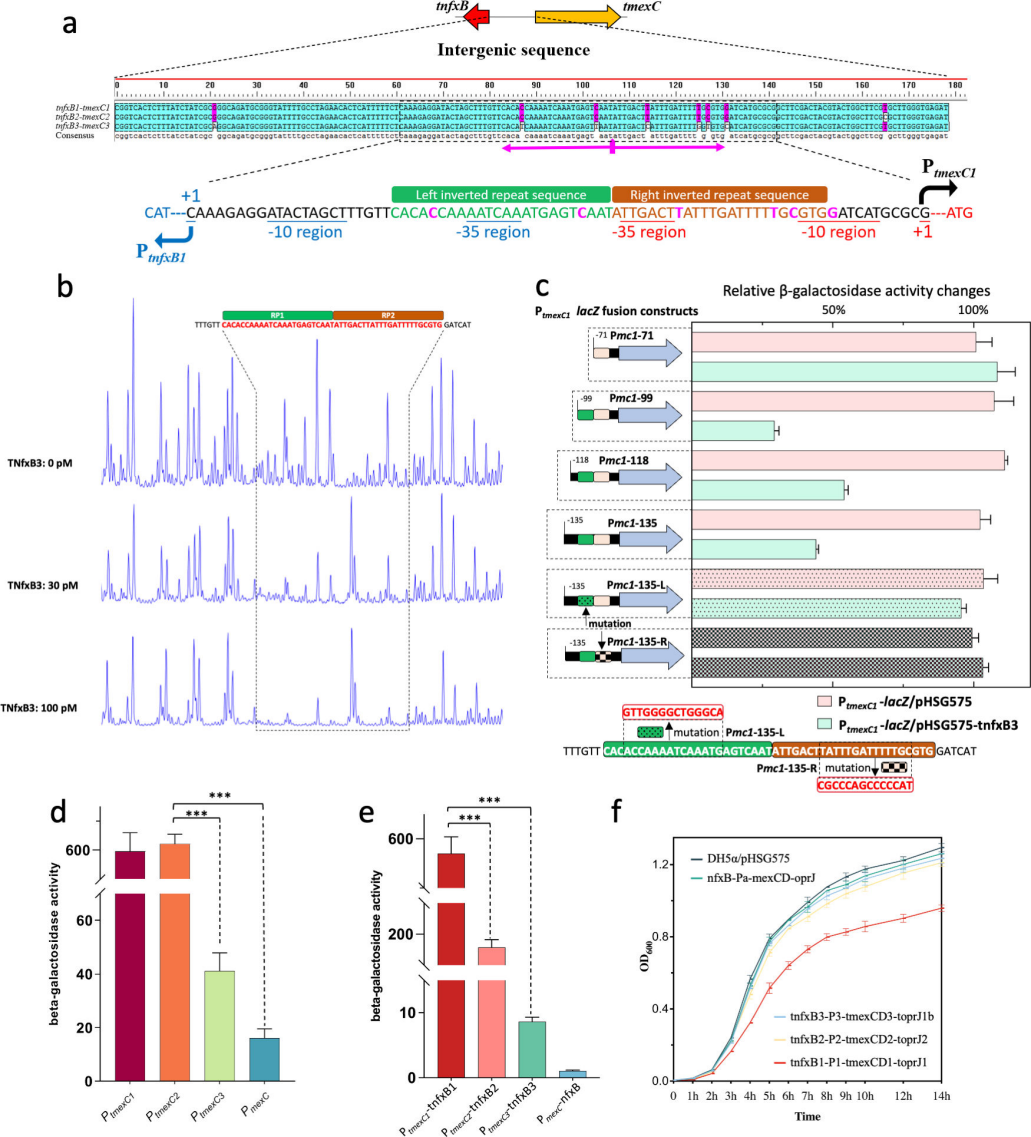
Identification of TNfxB3 DNA-binding site and the comparison of P*_tmexC_* and P*_mexC_* promoter strength. (**a**) Nucleotide sequence alignment of the intergenic region of *tnfxB-tmexC*. The transcription start sites of *tmexC* and *tnfxB* together with their promoter region were marked. (**b**) DNase I footprinting of TNfxB3 binding site at tnfxB1-tmexC1 intergenic sequence. The DNA fragment of FAM-labeled 200 bp tnfxB1-tmexC1 was incubated with two TNfxB3 protein concentration gradients. A reduction region in the intensity of DNase I-digested fragment was indicted box. The DNA sequence of the TNfxB3-protected region is shown above. (**c**) Relative β-galactosidase activity of a series of different-sized P*_tmexC1_-lacZ* fusion reporters coexpressed with pHSG575-tnfxB3 in *E. coli* strains compared with that with the empty vector. Fusion constructs contain 71 bp upstream of *tmexC1* ATG start codon (Pmc1-71), or progressively longer fragments of 99 bp P*_tmexC1_* (Pmc1-99), 118 bp (Pmc1-118), 135 bp (Pmc1-135), and P1-135 motif mutation (Pmc1-135-L, Pmc1-135-R). Two P1-135 motif mutations were constructed by mutating 14 bp A/T into C/G in each of the two inverted repeats regions. The schematic diagram of each of the cloning locations of upstream P*_tmexC1_* was depicted. Each bar represents the mean from three biological replicates. Each construct in the absence of TNfxB3 (empty vector) was used as a measure of basal promoter expression level. (**d**) The promoter activity of the three *tmexC* promoters and *mexC* was detected by the β-galactosidase analysis. (**e**) The β-galactosidase assay of the four *lacZ* fusion products under the regulation of the corresponding TNfxB or NfxB genes. (**f**) Growth curves of the five *E. coli* recombinant strains with efflux pump operon or empty plasmid. All experiments were performed three times, and data were displayed as mean ± SD. *P* values were determined by unpaired *t* test (*P < 0.05, **P < 0.01, and ***P < 0.001).

As shown above, TNfxB3 can bind to the intergenic sequence of *tnfxB-tmexC*, which may overlap with the promoter region of *tmexC*. Therefore, the transcription start sites of *tmexC*, together with the non-canonical −35 box and −10 elements of the two genes, were determined (Fig. 4a; Fig. S11). Interestingly, two 24-bp-long inverted repeat (IR) sequences were identified in the promoters of *tmexC* and *tnfxB* (Fig. 4a). To precisely localize the TNfxB3-binding sites, we performed a DNase I footprinting experiment using a 200-bp DNA fragment upstream of the *tmexC1* start codon. These two IRs were identified as the TNfxB3 protection region (Fig. 4b) and were accompanied by an unexpected central peak. In contrast, no obvious protective regions were found after incubation with the TNfxB3^T39R^ protein (Fig. S12). To further verify whether these two IRs are important for TNfxB3 interactions, a series of differently sized P*_tmexC1_-lacZ* fusion reporters were generated, including 71 bp upstream of the *tmexC1* ATG start codon (P*mc1*-71); progressively longer P*_tmexC1_* fragments of 99 bp (P*mc1*-99), 118 bp (P*mc1*-118), and 135 bp (P*mc1*-135); and P1-135 motif mutations (P*mc1*-135-L and P*mc1*-135-R). The generated fusion products were individually coexpressed with pHSG575-tnfxB3 or pHSG575 in *E. coli* (Fig. 4c). The presence of TNfxB3 in P*mc1*-71 did not change compared with that in the vector strain (Fig. 4c). In contrast, the expression of TNfxB3 in P*mc1*-99, P*mc1*-118, and P*mc1*-135 resulted in 75–50% decreases in β-gal activity relative to the control strain (Fig. 4c). After mutating 14 bp of A/T into C/G in one of the two IR regions (Fig. 4c), the decreased β-gal activity effect was lost (Fig. 4c). EMSA demonstrated that these DNA mutations diminish TNfxB3–DNA binding affinity (Fig. S13). These results suggest that TNfxB3 functions by binding to two IRs that overlap with the promoter region of *tmexCD-toprJ*.

Furthermore, because of the left IR sequences covering the −35 region of P*_tnfxB_* (Fig. 4a), we investigated the autoregulatory activity of TNfxB3. Consequently, the normal expression of *tnfxB3* under its promoter caused an obvious decrease in the LacZ activity of P*_tnfxB3_-lacZ*, whereas no change was observed in the presence of TNfxB1 (Fig. S14), demonstrating the negative autoregulatory function of TNfxB3.

### Weak TNfxB regulation coupled with high promoter activity contributes to MDR

Among the currently reported *tnfxB-tmexCD-toprJ* operons, differences exist not only in the TNfxB protein sequences, but also in the intergenic sequences of *tmexC-tnfxB* (Fig. 4a). To test whether these nucleotide differences would influence the expression of efflux pump genes, the corresponding promoter–*lacZ* fusions were constructed and expressed in *E. coli* DH5α. The β-gal results showed that P*_tmexC1_* and P*_tmexC2_* possessed the highest promoter activity, followed by P*_tmexC3_*, and P*_mexC_* showed the weakest activity (Fig. 4d; Fig. S15). To assess the joint effect of TNfxB regulation and the promoter region on the expression of the efflux pump, each regulator, *tnfxB* or *nfxB*, was coupled with the corresponding promoter–*lacZ* fusion constructs. The results revealed decreasing β-gal activity in the following order: P*_tmexC1_-tnfxB1* > P*_tmexC_-tnfxB2* > P*_tmexC_-tnfxB3* > P*_mexC_-*nfxB (Fig. 4e). These results were consistent with those of strains carrying P*_tmexC1_-tnfxB1-tmexCD1-toprJ1*, which exhibited high multidrug MIC levels relative to those of P*_tmexC3_-tnfxB3-tmexCD3-topJ1b* and P*_mexC_-nfxB-mexCD-oprJ* (Table 1).

Subsequently, to assess the effect of the expression of the four operons on bacterial fitness, we measured bacterial growth curves. The expression of *tnfxB1-tmexCD1-topJ1* significantly slowed the growth of the host strain for 14 h (Fig. 4f). Although expression of the other three efflux pumps led to slightly retarded growth, transformants carrying P*_mexC_-nfxB-mexCD-oprJ* showed growth similar to the control group (Fig. 4f), indicating that the expression levels of these efflux operons were positively correlated with the fitness burden on bacterial growth. Taken together, our data suggest that, although the expression of *tmexCD1-toprJ1* poses a bacterial growth burden, strong promoter activity and a weak TNfxB1 regulatory effect increase the expression of *tmexCD1-toprJ1*, leading to MDR in the host strains.

### Global dissemination of *tnfxB-tmexCD-toprJ*

**Fig 5:**
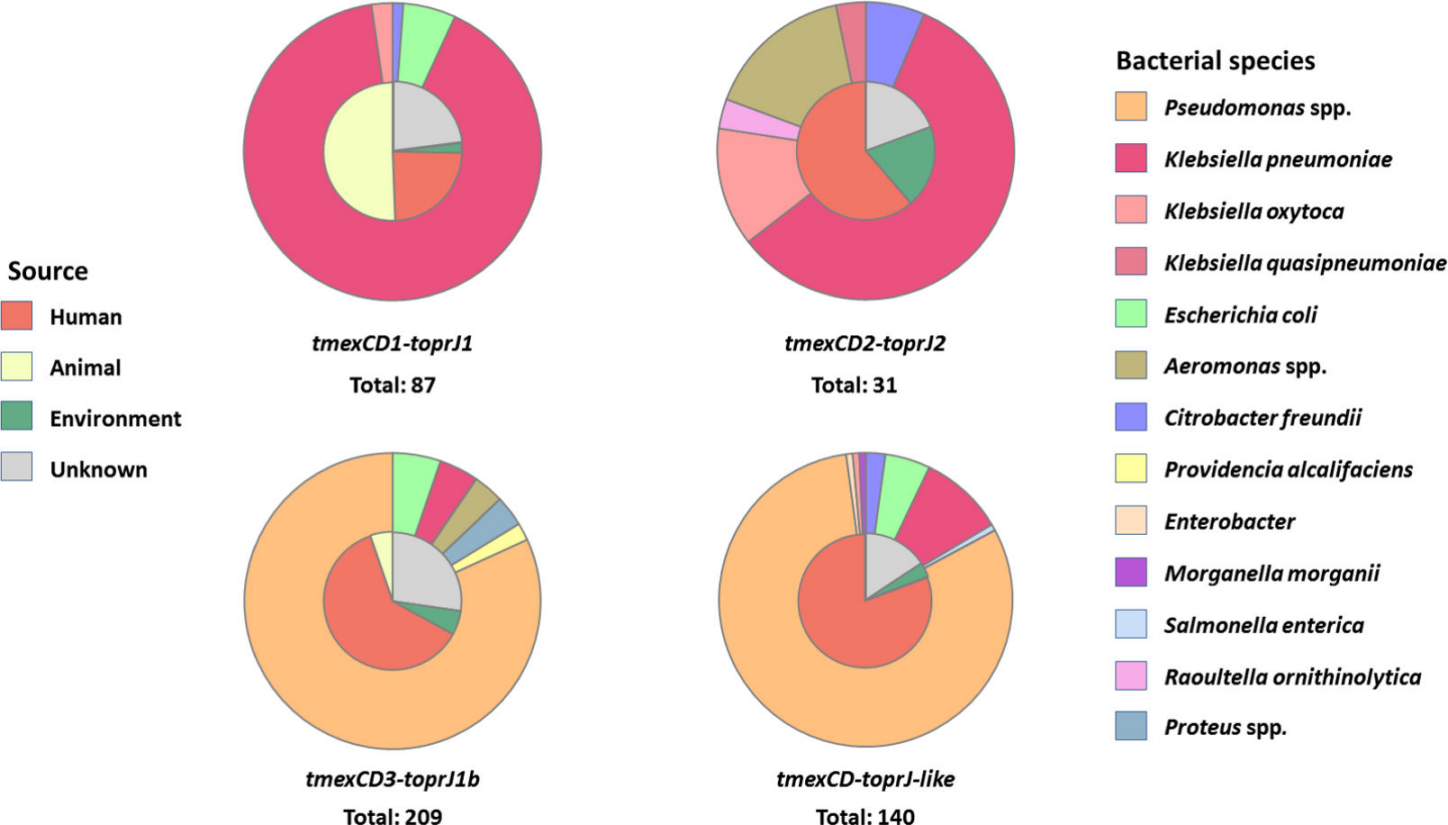
Global dissemination of *tmexCD-toprJ*. Isolation sources and bacterial species of organisms carrying different *tmexCD-toprJ* variants. All data were obtained from NCBI database. The inner and outer of the pie charts represent the isolation sources and the bacterial species of *tmexCD-toprJ*-positive strains, respectively.

To analyze the current distribution of *tnfxB-tmexCD-toprJ*, we collected information on all independent isolates carrying *tnfxB-tmexCD-toprJ* deposited in the GenBank database. Various variants of *tnfxB-tmexCD-toprJ* are emerging and spreading globally (Fig. S16). A total of 467 strains were obtained, of which 282 were isolated from human clinics. While 236 *tnfxB-tmexCD-toprJ*-positive isolates were obtained from China, *tnfxB-tmexCD-toprJ* was also detected in 171 strains isolated from 30 other countries (Fig. S16). Except in China, where *tnfxB1-tmexCD1-toprJ1* was dominant, *tnfxB3-tmexCD3-toprJ1b* and *tnfxB-tmexCD-toprJ*-like gene clusters were the predominant *tnfxB-tmexCD-toprJ* gene clusters in most countries (Fig. S16). Moreover, *tmexCD1-toprJ1* and *tmexCD2-toprJ2* were mostly identified in *Klebsiella* (93% and 74%, respectively), whereas *tmexCD3-toprJ1b* and other *tmexCD-toprJ*-like gene clusters were mainly observed in *Pseudomonas* (83% and 81%, respectively). Human clinical *Pseudomonas* is the primary host bacterium of *tmexCD-toprJ-*like gene clusters (Fig. 5a).

## DISCUSSION

Horizontally acquired ARGs drive the rapid occurrence and evolution of AMR (22). These mobile determinants commonly originate from intrinsic chromosomal proto-resistance genes with physiological functions (23). Some ARG progenitors confer the phenotypic activity of AMR, while several do not mediate drug resistance in their native context (24, 25); for example, chromosomal MexCD-OprJ is functional but barely expressed. Based on our comparative analysis of the three *tnfxB-tmexCD-toprJ* and *nfxB-mexCD-oprJ* operons, we revealed that plasmid-borne TMexCD1-TOprJ1 mediates higher MDR than its ancestor, MexCD-OprJ. We discovered that key sequence alterations in both the local repressor and promoter regions cooperatively led to a higher expression level of *tmexCD1-toprJ1* and a higher resistance level of the host strain. Remarkably, weakening repression regulation and strengthening promoter expression levels were clearly observed from *nfxB-mexCD-oprJ* to *tnfxB3-tmexCD3-toprJ1b* and then to *tnfxB1-tmexCD1-toprJ1*, outlining a distinct evolutionary feature of mobile ARGs compared to their precursors.

NfxB belongs to the TFRs (21), which have been widely associated with bacterial physiology aspects (26). In clinical isolates, TFR mutations lead to the de-repression of their targets (27), some of which are efflux transporters, thus contributing to AMR and adjusting to changing environments (28). NfxB mutants are commonly found in ciprofloxacin-resistant *P. aeruginosa* clinical strains (29, 30). Previous structural studies on several TFRs have revealed that the arginine residue in the HTH domain is in direct contact with the DNA phosphate backbone (31). In this study, we found that the T39 residue in the HTH domain of TNfxB/NfxB is critical for its affinity for DNA, which explains its regulatory function in the expression of *tmexCD-toprJ*. T39 is highly conserved among TNfxB-like and NfxB homologs. However, a T39R substitution occurs in TNfxB1. This substitution weakens its repressor function, resulting in increased expression of the *tmexCD1-toprJ1* efflux pump and enhanced MICs for tigecycline and other antimicrobial agents. This could be because arginine possesses a long and positively charged side chain; its interposition seems to change the relative conformation and affect the stable binding pose between the threonine in TNfxB and the DNA (Fig. S17).

Although the 39th residue in TNfxB3 and NfxB is threonine, our data revealed that TNfxB3 mediates a weaker downregulation effect than its progenitor NfxB. This is because TNfxB3 exhibits a relatively lower operator DNA-binding ability than the progenitor NfxB. Furthermore, NfxB expression was significantly higher than TNfxB3 expression. In addition to transcription, protein synthesis and degradation are involved in cellular protein production (32). Bacterial proteins commonly carry multiple post-translational modifications (33), some of which might modulate protein stability; for example, acetylation of HilD benefits its stability in *Salmonella Typhimurium* (34). We speculate that different post-translational modifications may occur in TNfxB and NfxB, influencing their protein stability. This warrants further investigation.

In addition to transcription factors, the promoters of ARGs affect their transcription efficiency, leading to different drug resistance phenotypes (35). Previous studies indicate that several mutations in core promoter regions influence ARG-mediated resistance levels (36). For instance, promoter mutations in the *mtrCDE* membrane pump operon enhance efflux activity in *Neisseria gonorrhoeae* (37). Strong and weak promoters can be classified according to their relative strength. Our molecular analysis revealed that both P*_tmexC1_* and P*_tmexC2_* possess strong promoters, whereas P*_mexC_* and P*_tmexC3_* promoters are relatively weak. Additionally, TNfxB3 downregulated the effect of *tmexCD-toprJ* while TNfxB1 did not. Therefore, in the *tnfxB1-tmexCD1-toprJ1* operon, the weak regulatory function of TNfxB1 and the strong promoter function of P*_tmexC1_* work together to shape the high-level MDR phenotype mediated by the overexpression of *tmexCD1-toprJ1*. Moreover, this explains the medium-level antibiotic resistance phenotypes mediated by TNfxB3-TMexCD3-TOprJ1. Although residue T39 also exists in TNfxB2, *tnfxB2* gene is generally embedded by the insertion sequence element resulting in the loss of promoter and starting amino acids, which cooperates with the strong promoter P*_tmexC2_* to create a higher transcription-level version of *tmexCD2-toprJ2*, similar to *tmexCD1-toprJ1*. This illustrates that bacteria can evolve through multiple mechanisms to adapt to changing circumstances, such as antibiotic pressure.

Epidemiological analysis of GenBank data showed that Enterobacteriaceae are the primary carriers of the high-level MDR gene clusters *tnfxB1-tmexCD1-toprJ1* and *tnfxB2-tmexCD2-toprJ2*, whereas the major host reservoirs for *tnfxB3-tmexCD3-toprJ1b* are *Pseudomonas* spp., which are the source of the *nfxB-mexCD-oprJ* operon. Previous studies have also revealed similar prevalence characteristics of *tmexCD-toprJ* variants (16, 17). These epidemiological features of *tmexCD-toprJ* variants may be attributed to the trade-off between the benefits of these ARGs and the cost of their expression. The fitness costs accompanied by the expression of these transporter proteins are commonly the results of the energy expense and constant extrusion of the metabolic substrates by the activity of the pumps in the absence of antibiotics (38–40). Because efflux pump expression is costly and unnecessary under no-antibiotic conditions, in the chromosomal *nfxB-mexCD-oprJ* operon, the MexCD-OprJ efflux system is silent under the tight negative control of NfxB. However, during drug exposure, NfxB mutants have emerged to increase chromosomal efflux pump-mediated resistance in clinical isolates and experimental evolution (41, 42). In this study, we observed that the weak regulation mode resulted in a higher AMR level for the expression of plasmid-encoded *tmexCD1-toprJ1*, which is prevalent in farm animal-associated isolates that are exposed to high drug-induced selective pressure, such as tetracycline exposure (43). Although the high expression level of these transporter proteins imposes a substantial fitness burden on the host bacteria, simultaneously, the overexpression of the *tmexCD1-toprJ1* determinant provides the host bacteria with survival capacity under drug-selective pressure, which further promotes its spread in Enterobacteriaceae. However, the *tnfxB3-tmexCD3-toprJ1* variant which confers only moderate MDR level and relatively low fitness burden, was limited to *Pseudomonas*. This suggests that, if the acquisition of foreign resistance genes can no longer provide sufficient drug resistance to the host bacteria, it becomes difficult to maintain their existence. Hence, the expression and epidemiological features of *tmexCD-toprJ* variants illustrate the critical role of antibiotic-selective pressure in driving the evolution and spread of ARGs. Because of the fitness cost posed by *tmexCD-toprJ*, it is possible that decreasing the use of antimicrobial agents in the farm industry would reduce the incidence of *tmexCD1-toprJ1*, similar to the decrease in *mcr-1* observed in animals after colistin withdrawal (44). Our findings support drug-selective pressure as a driving force of ARG evolution and spread; therefore, further efforts, including rational drug use, are needed to prevent the emergence of more novel ARGs in clinical pathogens in the future.

Efflux pumps participate in various aspects of bacterial physiology except for their role in antimicrobial resistance, such as bacterial quorum sensing (QS) response (5, 45). The overexpression of MexCD-OprJ system in *nfxB* mutants was reported to impact the virulence of *P. aeruginosa* host cells owing to extruding QS signal substrates, such as kynurenine (46, 47). The major *tmexCD-toprJ* variant carried by *Pseudomonas* was *tmexCD3-toprJ1b* (Fig. 5). Although the natural substrate of TMexCD3-TOprJ1 system remains unknown, the expression of TMexCD3-TOprJ1 under the relatively weak regulation of TNfxB3 potentially impact the bacterial quorum sensing and virulence-related phenotypes in *Pseudomonas*, which deserves further investigation.

**Fig 6:**
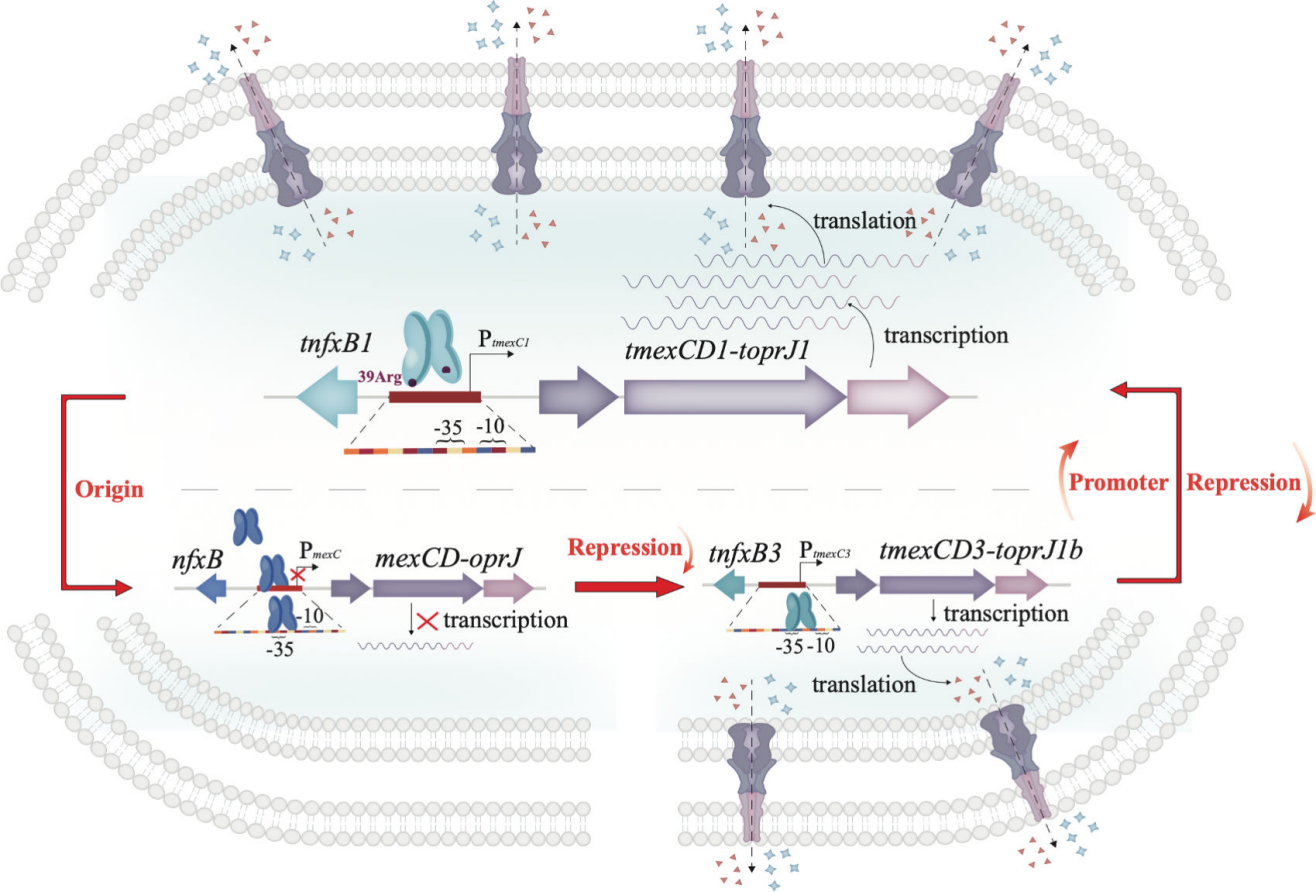
Schematic diagram of regulation models of *tmexCD-toprJ* operons and its origin, *mexCD-oprJ*. In inactive operon, *mexCD-oprJ*, was silent owing to the existence of repressor NfxB. In the intermediate state, *tnfxB3-tmexCD3-toprJ1b* operon, TNfxB3 with weaker DNA binding ability and less protein production partly relieved the repression of *tmexCD-toprJ* and conferred low-level antibiotic resistance. In the currently prevalent version, *tnfxB1-tmexCD1-toprJ1*, T39R mutation significantly reduces the DNA binding affinity to P*_tmexC1_* region, and *tmexC* the promoter activity of *tmexC1* gets stronger, which results in more efflux pump creation and confers higher multidrug resistance level.

In summary, our study provides a comprehensive understanding of the progressive evolution of the MDR determinant *tmexCD1-toprJ1* through complex regulatory mechanisms and promoter activity. In the progenitor state, *mexCD-oprJ* exists as a silent operon barely contributing to antibiotic resistance, because of both the strong repressor NfxB and low promoter activity. However, in *tnfxB1-tmexCD1-toprJ1*, the most prevalent version in Enterobacteriaceae, a single amino acid variant (T39R) in TNfxB1 significantly reduced the DNA-binding affinity to the P*_tmexC1_* region, and the promoter activity of *tmexC1* increased. Together, both critical factors elevated the transcription of *tmexCD1-toprJ1*, aggravating MDR by producing more efflux pump machines (Fig. 6). This study explores the evolutionary dynamics of antimicrobial resistance development and offers insight into the design of potential inhibitors to reinforce the transcriptional repression of ARG to combat AMR.

## MATERIALS AND METHODS

### Bacterial strains, plasmid construction, and determination of susceptibility to antimicrobial agents

The bacterial strains and plasmids used in this study are described in Table S3. Recombinant plasmids and site-directed mutagenesis were constructed using a seamless assembly cloning kit (Clone Smarter) with the primers listed in Table S4. The constructs were confirmed using PCR and Sanger sequencing. The MICs of these strains against five antibiotics (tigecycline, tetracycline, cefquinome, cefepime, and streptomycin) were determined by broth dilution or agar dilution assays according to Clinical and Laboratory Standards Institute guidelines (48). *E. coli* ATCC 25922 served as the reference strain for antimicrobial susceptibility testing.

### RNA extraction and quantitative RT-PCR

The total RNA of the recombinant strains was extracted using the TRIzol method with a HiPure Bacterial RNA Kit (Magen, China) according to the manufacturer’s instructions. A NanoDrop spectrophotometer (Thermo Fisher Scientific, Waltham, MA, USA) and agarose gel electrophoresis were used to assess the concentration and quality of RNA, and cDNA was synthesized using an RNA Reverse Transcription Kit (TsingKe Biotech, China). qPCR was performed to measure the expression levels of efflux pump genes using the Tsingke Master qPCR Mix (TsingKe Biotech, China) with primers (Table S4) and the 16S rRNA gene as an internal reference. Based on a previous report, the 2^△△–*Ct*^ method was used to analyze the relative expression values (49).

### β-galactosidase assay

This assay was conducted based on previous studies (50). Briefly, bacterial cells were cultured overnight, harvested, and suspended in a lysis buffer. The bacterial cells were lysed using a Q500 Sonicator (Qsonica L.L.C, USA). The lysates were centrifuged, and the supernatants were used to determine protein concentration and enzyme activity. The protein concentration was measured using a Bradford protein assay kit (Takara, Japan). The β-gal enzyme in the supernatants and 200 µL of 4 mg/mL O-nitrophenyl-β-D-galactopyranoside were reacted at 30°C, then the absorbance was measured at 420 nm using an EnSight Multimode Microplate Reader (PerkinElmer). The enzyme activity was calculated according to the formula described by Yang et al. (51).

### Rapid amplification of cDNA ends (RACE)

The transcriptional start sites of *tnfxB1* or *tmexC1* were determined based on a 5′ RACE kit (Sangon Biotech). Briefly, the first-strand cDNA was synthesized from the extracted total RNA of *E. coli* DH5α/pHSG575-tnfxB1-tmexCD1-toprJ1 using specific 5′ RACE primers. The RNA template was then removed using RNase H. The cDNA was further oligo-dC-tailed, and two rounds of PCR amplification were conducted to generate sufficient specific products to perform Sanger Sequencing and match the transcriptional start sites.

### Western blot assay

C-terminal FLAG-tagged *tnfxB* or *nfxB* genes were cloned into pHSG575 and transformed into *E. coli* DH5α for the western blot analysis. Bacterial cells (30 mL) from the early exponential phase (OD_600_ = 0.3) were harvested by centrifugation at 4°C and subsequently resuspended in 2 mL phosphate-buffered saline (PBS). The resuspension was subjected to sonication, followed by 20 min of centrifugation at 4°C to collect the supernatant. The protein concentrations were determined using an Enhanced BCA Protein Assay Kit (Beyotime, Shanghai, China). For each strain, the same amount of protein (40 µg) was loaded onto 12.5% sodium dodecyl sulfate-polyacrylamide gel electrophoresis (SDS-PAGE) gels and transferred onto nitrocellulose membranes. To detect target proteins, the membranes were incubated with the corresponding antibodies, followed by treatment with SuperSigna West Pico PLUS Chemiluminescent Substrate (Thermo Fisher Scientific). The membranes were exposed to the Azure C200 Gel Imaging System (Azure Biosystems, USA), and the western blot bands were quantified using ImageJ software. GAPDH was used as the loading control for each assay. The primary antibodies used were anti-GAPDH (BioLegend, San Diego, CA, USA) and anti-FLAG (Abcam, Cambridge, UK).

### Expression and purification of His-tagged TNfxB and NfxB proteins

Open-read fragments of *tnfxB* or *nfxB* were cloned into pMAL-c5x with a 6 × His tag. The recombinant plasmid was then introduced into *E. coli* BL21(DE3) pLysS and cultured in 500 mL Luria-Bertani broth at 30°C with shaking, until the OD_600_ reached 0.4–0.5. Then, 0.25 mM isopropyl β-D-1-thiogalactopyranoside was added to induce the TNfxB-His or NfxB-His protein, and the cells were cultured for 20 h at 16°C. The bacterial cells were harvested by centrifugation at 5,000 × *g* for 20 min and the pellets were resuspended in 50 mM phosphate buffer (pH 7). The harvested cell suspension was split by a low-temperature ultra-high pressure cell disrupter (JN-2.5C, Juneng Biol) and was further centrifuged at 4°C, 13,000 × *g*, for 20 min. The supernatant was filtered through a 0.45-µm filter and the His-tagged protein was enriched and purified using an AKTA pure chromatography system (Cytiva) with a HisTrap HP purification column. Finally, desalting columns were used to remove the salts from proteins, which were stored at 4°C. Protein concentrations were determined using a bicinchoninic acid assay (Thermo Fisher Scientific). Protein purity was verified using SDS-PAGE on a 12.5% polyacrylamide gel.

### Electrophoretic mobility shift assay

This assay was conducted using a Thermo EMSA kit (Thermo Fisher Scientific). Briefly, the DNA fragment containing the intergenic sequence between *tnfxB1* and *tmexC1* was amplified by PCR using the primers listed in Table S4. Binding reactions were performed in 20 µL volumes of PBS buffer (137 mM NaCl, 2.7 mM KCl, 8 mM Na_2_HPO_4_, and 2 mM KH_2_PO_4_), containing 2 µL glycerin, 80 ng DNA, and 5–70 pmol TNfxB or NfxB. After 30 min of incubation at 25°C, the reaction mixtures were resolved on a 10% acrylamide gel for native PAGE in tris-acetate-ethylenediaminetetraacetic acid buffer for 80 min at 120 V at 25°C. The gels were stained with GoldView nucleic acid staining dye and visualized using the Bio-Rad ChemiDoc XRS + system (Bio-Rad, CA, USA).

### DNase I footprinting assay

The intergenic region of *tnfxB1-tmexC1* was amplified by PCR using 6-FAM (5′ end) labeled primers. Then, 400 ng of this DNA probe was incubated with different amounts of TNfxB3 or TNfxB3^T39R^ proteins in a 50 µL volume. Incubation was continued for 30 min at 25°C, followed by adding 0.02 U DNase I and further incubation at 30°C for 5 min. The reaction was stopped by adding DNase I stop solution. DNA fragments were extracted from the mixed samples using phenol/chloroform. After being combined with centrifugation and ethanol, the DNA was further purified and dissolved in 10 µL distilled water. The samples were then analyzed using the terminal restriction fragment length polymorphism technique (Sangon Biotech) with GeneScan-500 LIZ size standards. The results were analyzed using PeakScanner V2.0 software (Thermo Fisher Scientific).

### Surface plasmon resonance

The interaction between TNfxB3 or NfxB and the operator DNA was further assessed using a Biacore X100 system (GE Healthcare). Biotinylated double-stranded DNA (81 bp, 10 µM) containing the two IRs was synthesized by Beijing Tsingke Biotech Co., Ltd. and immobilized on a streptavidin sensor chip (GE Healthcare) at a density of 500–600 resonance units. The TNfxB3 and NfxB proteins were serially diluted in PBS buffer at concentration ranges of 125–1500 nM and 62.5–1000 nM, respectively. The binding assay was performed at 25°C and a flow rate of 30 µL/min with PBS as the running buffer. The protein was injected and allowed contact with the DNA surface for 2 min, followed by 400 s of buffer flow to record the dissociation. Regeneration was performed using 10 mM Gly\HCl (pH 3) for 1 min, followed by 2 M MgCl_2_ for 1 min, and then brief washing with the buffer. The experimental data were corrected for nonspecific background binding curves obtained using the running buffer alone. The binding affinity (*K*_*D*_) was calculated using Biacore X100 evaluation software with the classical single-interaction (1:1) Langmuir model.

### Structure homology modeling

The structures of NfxB and TNfxB3 were generated by RosettaFold protein structure prediction (52). They were then aligned and the RMSD values were calculated using PyMOL. We used Web 3DNA 2.0 (http://web.x3dna.org) to predict the DNA 3D structures of the promoter region of *tmexC1* (53). Next, we determined the interaction between NfxB and its binding DNA using Discovery Studio and the ZDOCK docking algorithm (54). Threonine was selected as the binding sites of threonine in NfxB to generate a total of ∼2,000 binding poses that were more than 10 Å RMSD apart in the NfxB–DNA complex.

### Phylogenetic analysis of TNfxB homologs

A total of 95 TNfxB-like proteins with greater than 60% amino acid identity were selected and obtained from the GenBank database for alignment. MegaX was then used to construct a neighbor-joining phylogenetic tree (55), which was visualized using iTol (56).

### Statistical analyses

All experiments were performed in biological triplicates and data were presented as means ± SD. GraphPad Prism 6 was employed for statistical analysis. Two-tailed unpaired *t* test was used to calculate *P* values and significant differences (*, *P* < 0.05; **, *P* < 0.01; and ***, *P* < 0.001).

## SUPPLEMENTAL MATERIAL

Supplemental material mbio.00218-24-s0001.docx Fig. S1 to S17 and Tables S1 to S4.

ASM does not own the copyrights to Supplemental Material that may be linked to, or accessed through, an article. The authors have granted ASM a non-exclusive, world-wide license to publish the Supplemental Material files. Please contact the corresponding author directly for reuse.
