# Supplementary material for: Promoter regulatory mode evolution enhances the high multidrug resistance of tmexCD1-toprJ1
Source: mBio. 2024 Apr 2;15(5):e00218-24. doi: 10.1128/mbio.00218-24 (PMC11077950; doi:10.1128/mbio.00218-24)
Supplement: Supplemental material — Fig. S1 to S17 and Tables S1 to S4. [file mbio.00218-24-s0001.docx]

**Supplemental materials**

**Supplemental Figures:**

**
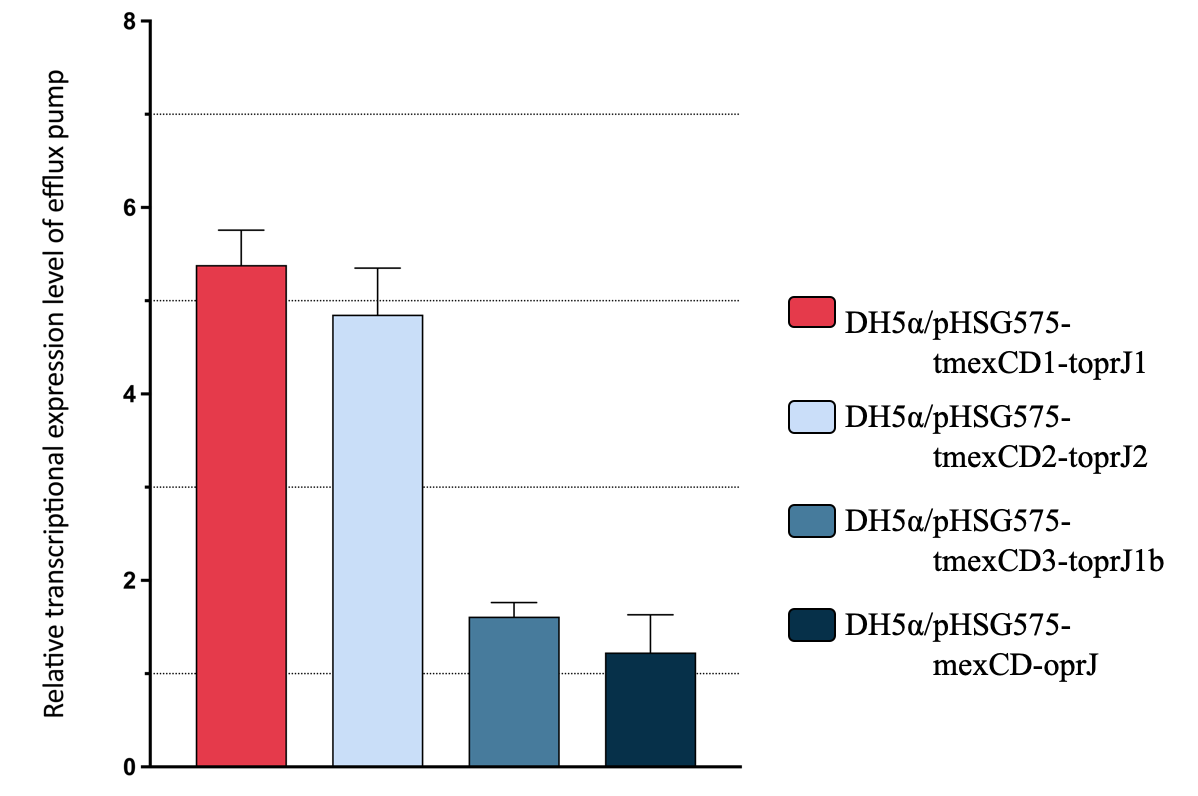
**

Fig. S1 Relative transcriptional expression level of efflux pump gene in four recombinant strains carrying four operons without their local regulator. The radio pf expression level was compared with strain DH5α/pHSG575-mexCD-oprJ. 16S rRNA gene was used to normalize the gene expression.


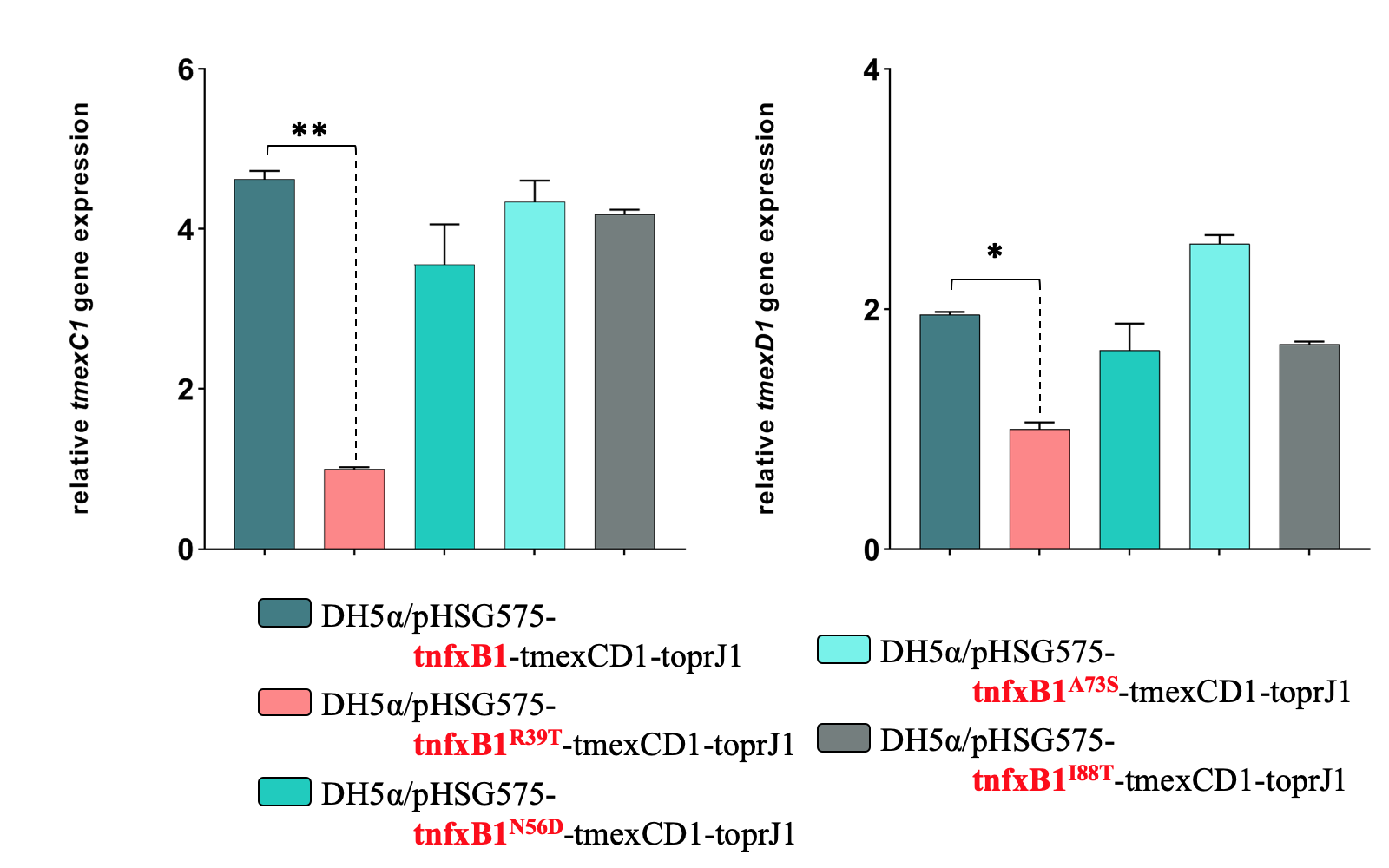


A

B


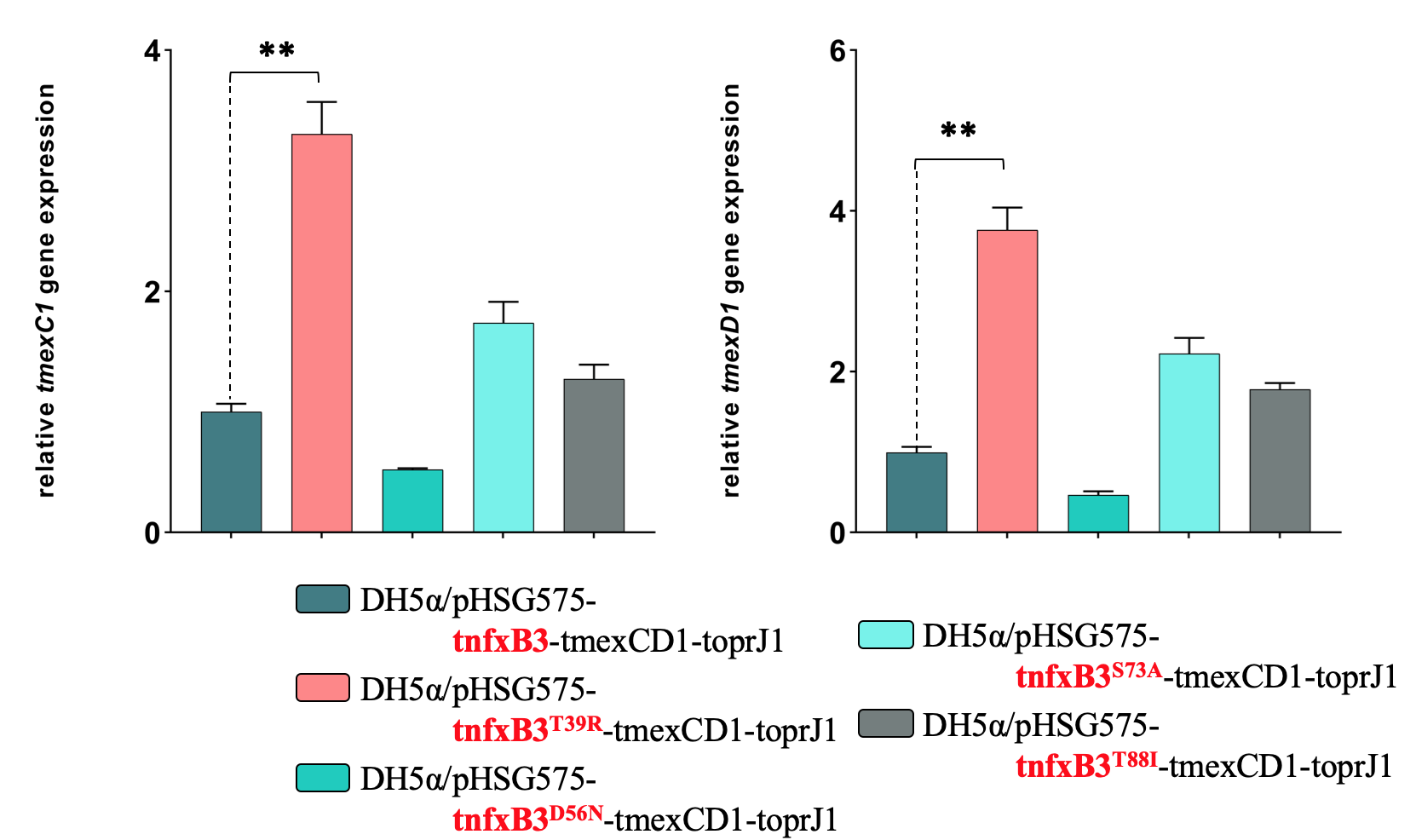


Fig. S2 Relative transcriptional expression level of *tmexC1* and *tmexD1* genes under the regulation of TNfxB mutants. (A) The effects of the single point mutation in TNfxB1 on the relative mRNA expression level of *tmexCD1* genes relative to that carrying *tnfxB1^R39T^*. (B) The effect of TNfxB3 mutation in threonine 39 on the expression of *tmexCD1* genes compared to that carrying wild-type TNfxB3.


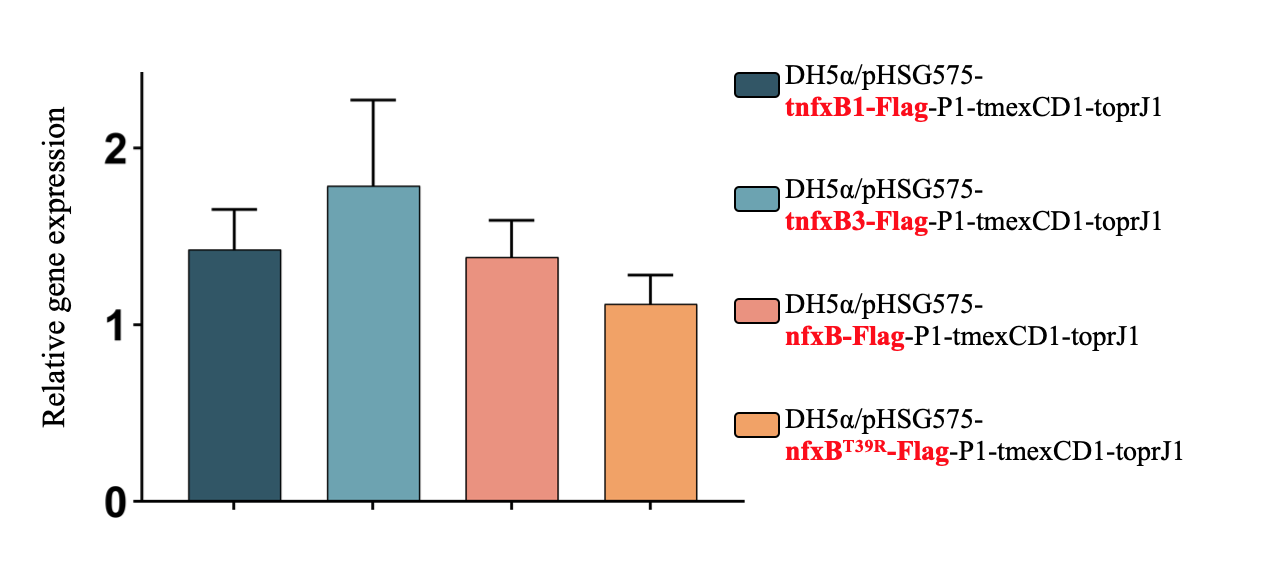


Fig. S3 Transcriptional expression level of FLAG-tagged TNfxB or NfxB or its mutants in recombinants relative to the same host strain expressing *nfxB^T39R^* detected by RT-qPCR.


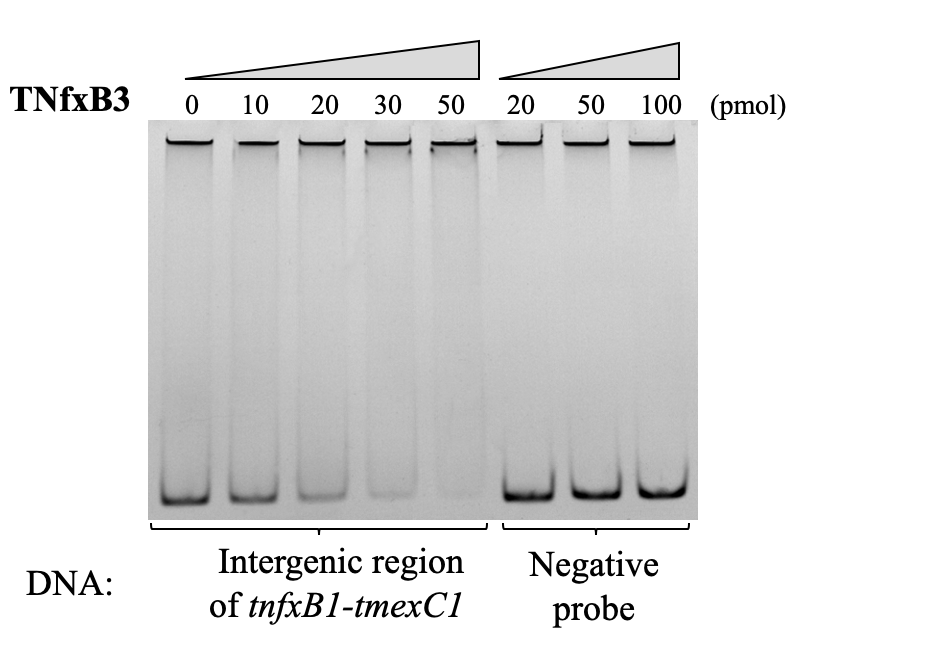


Fig. S4 EMSA analysis of TNfxB3 interaction with Intergenic DNA of *tnfxB1-tmexC1* or the negative DNA probe. The negative probe used here was a 200-bp *tmexD* gene sequence amplified by PCR.


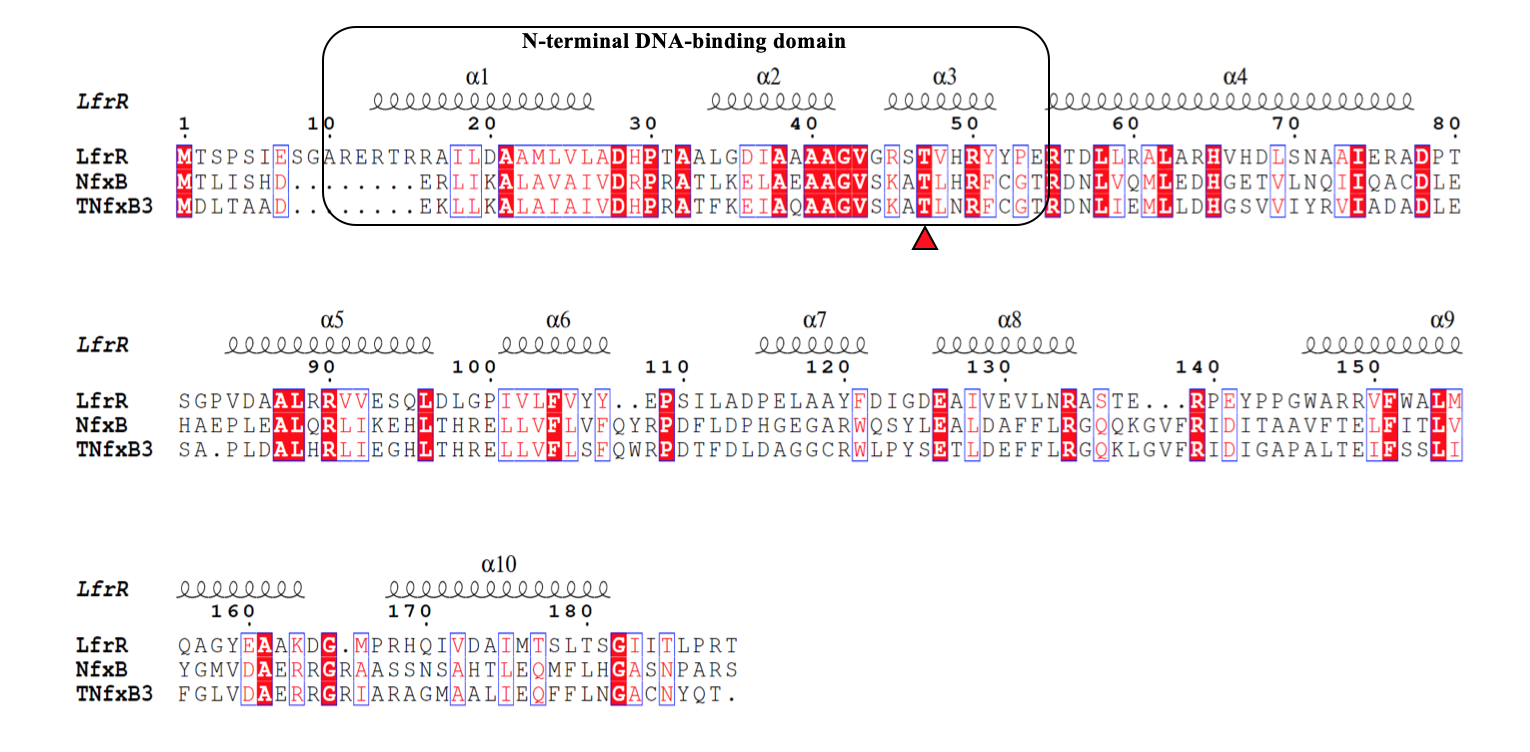


Fig. S5 Alignment of the amino acid sequences of LfrR (PDB number: 2V57), NfxB, and TNfxB3. The DNA binding domain was boxed and conserved Threonine 39 was indicated.


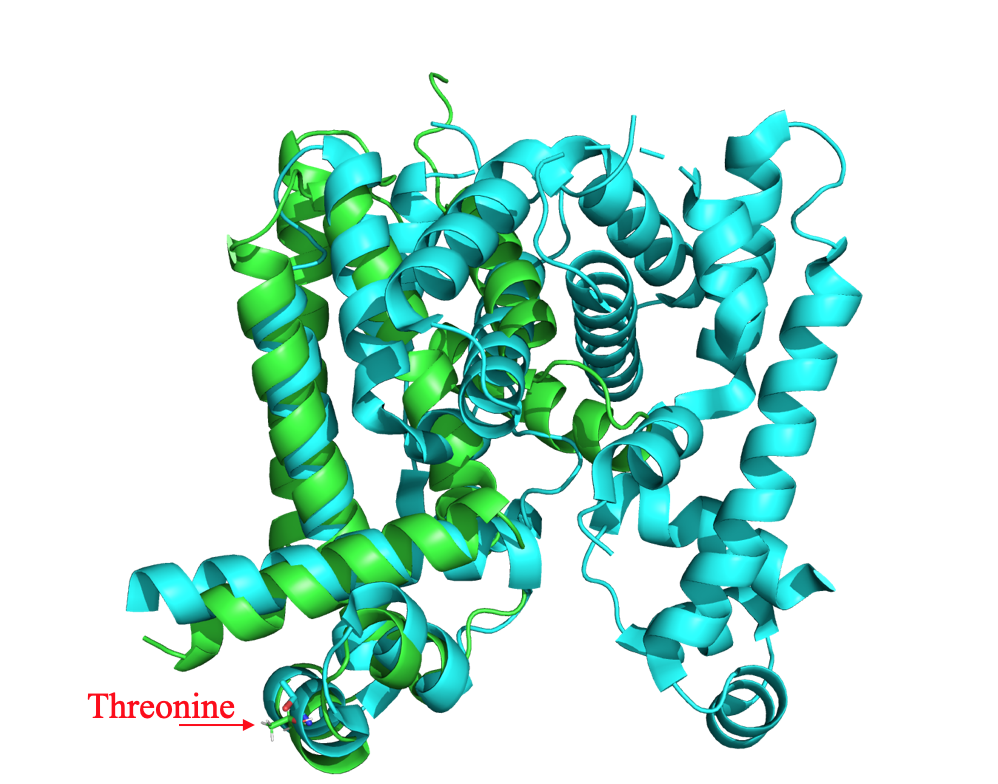


Fig. S6 Alignment of protein structure between TNfxB3 and the dimer of LfrR (PDB number: 2V57). Structures with blue and green colors represent LfrR, and TNfxB3, respectively. The conserved threonine in LfrR and TNfxB3 were indicated.


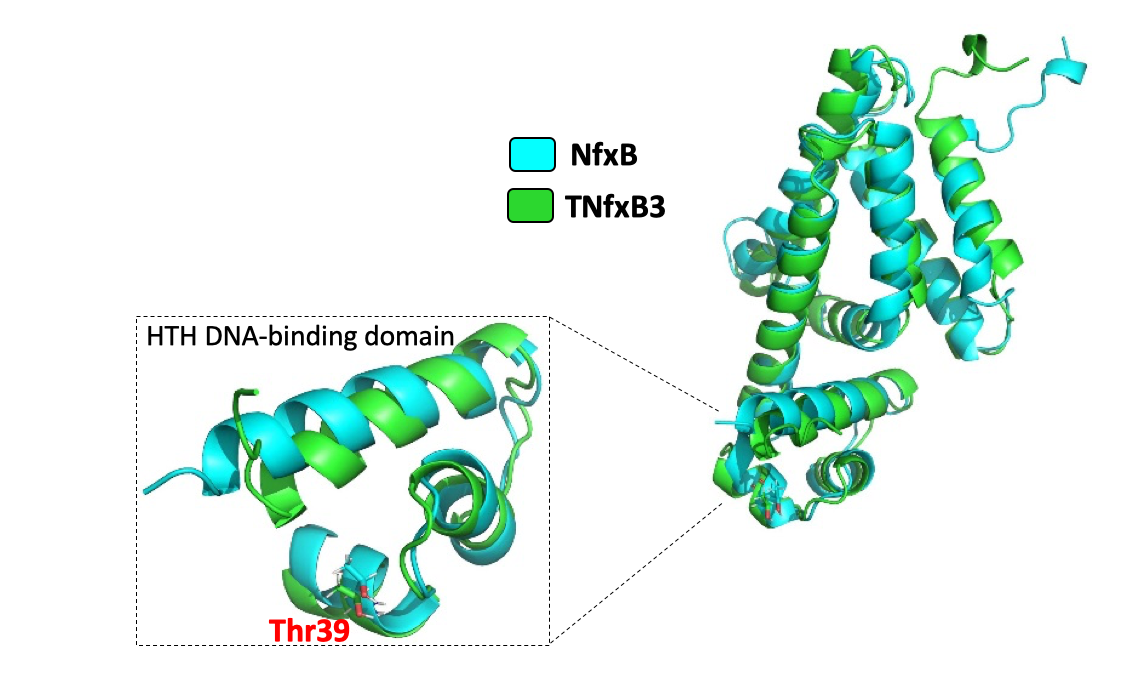


Fig. S7 The homology model comparison of NfxB and TNfxB3. Protein structures with the DNA binding domain were zoomed in. The residue 39 threonine in TNfxB3 and NfxB were visualized.


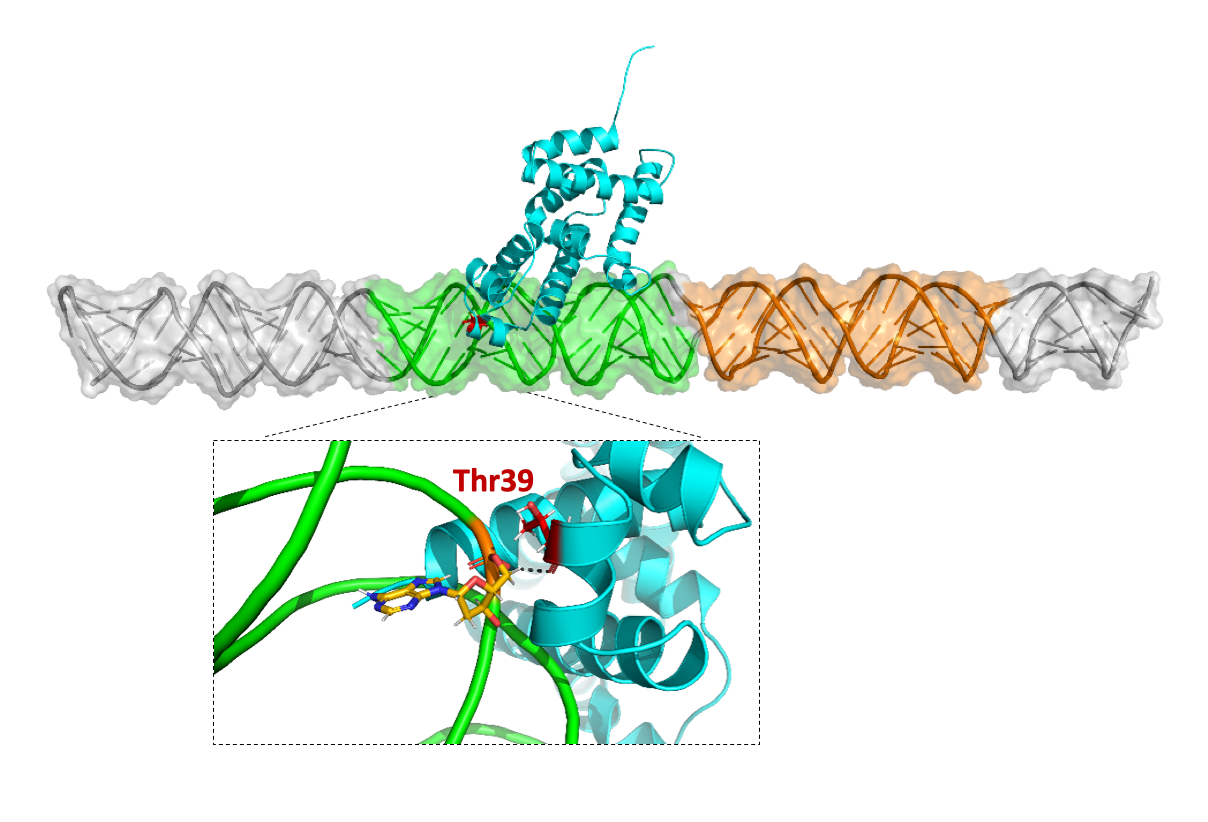


Fig. S8 Molecular docking analysis for the DNA binding of NfxB. DNA backbones containing inverted repeats are shown in green and brown, while NfxB monomer was visualized as blue. Dashed line represents the hydrogen bond interaction.


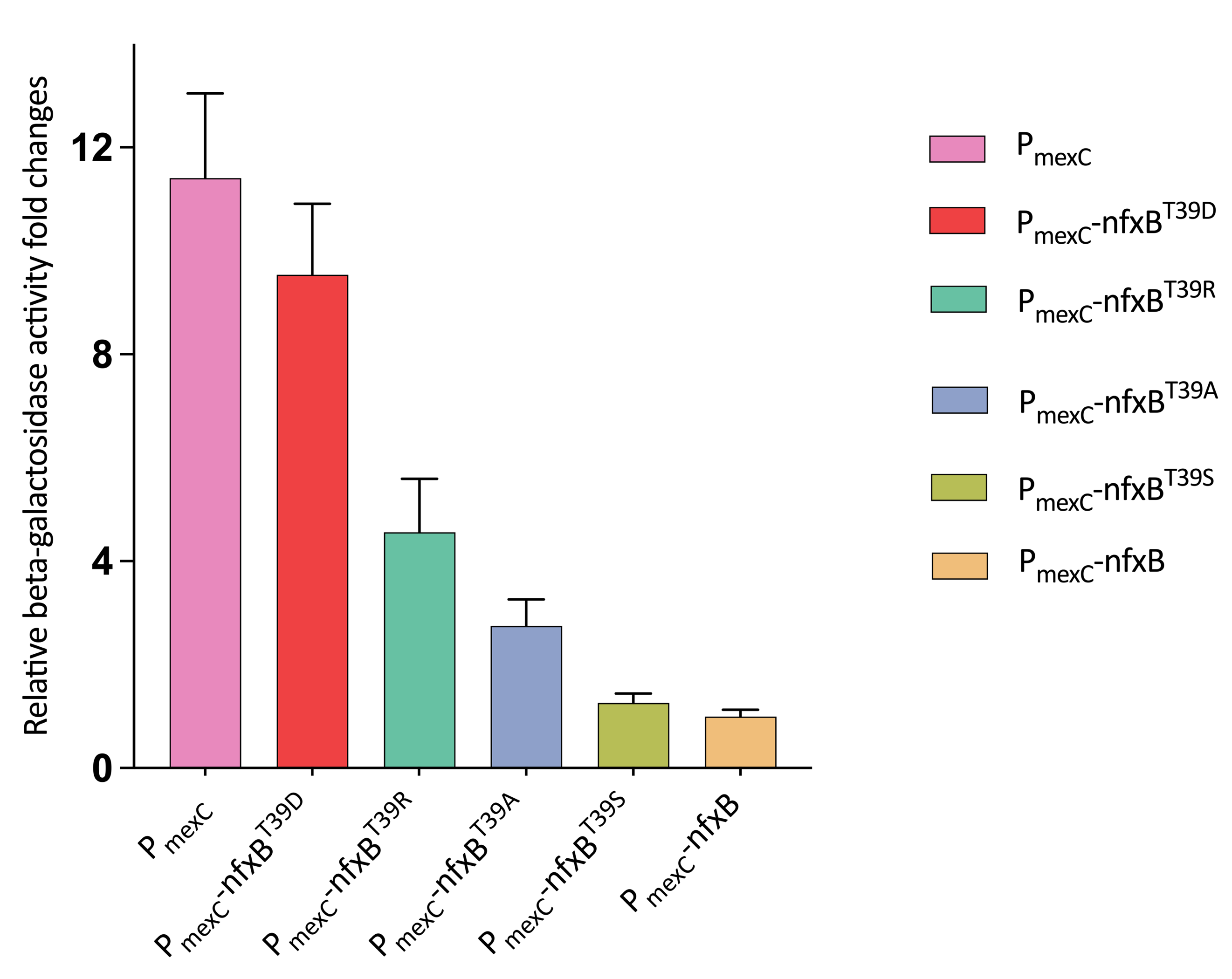


Fig. S9 Relative β-galactosidase activity fold changes of six P*_mexC_*-*lacZ* fusion products compared with that carrying P*_mexC_-nfxB*. The β-galactosidase activity of strain carrying fusion product of P*_mexC_-nfxB* was used as a measure of basal expression


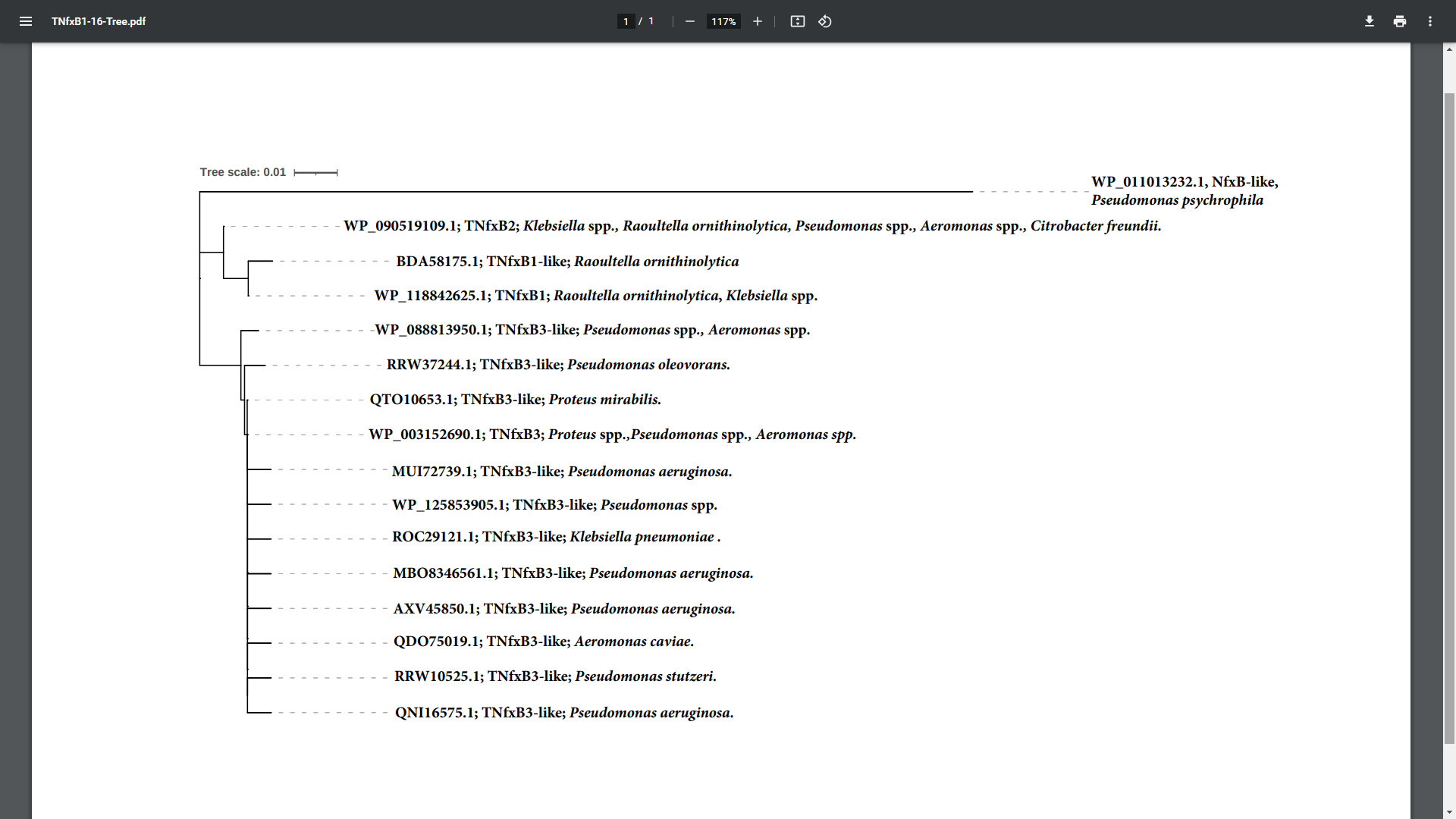


Fig. S10 A phylogenetic tree of TNfxB-like homologs. A total of 16 representative TNfxB homologs were collected to construct a neighbor-joining tree. The GenBank accession numbers and bacterial species of these proteins were shown.


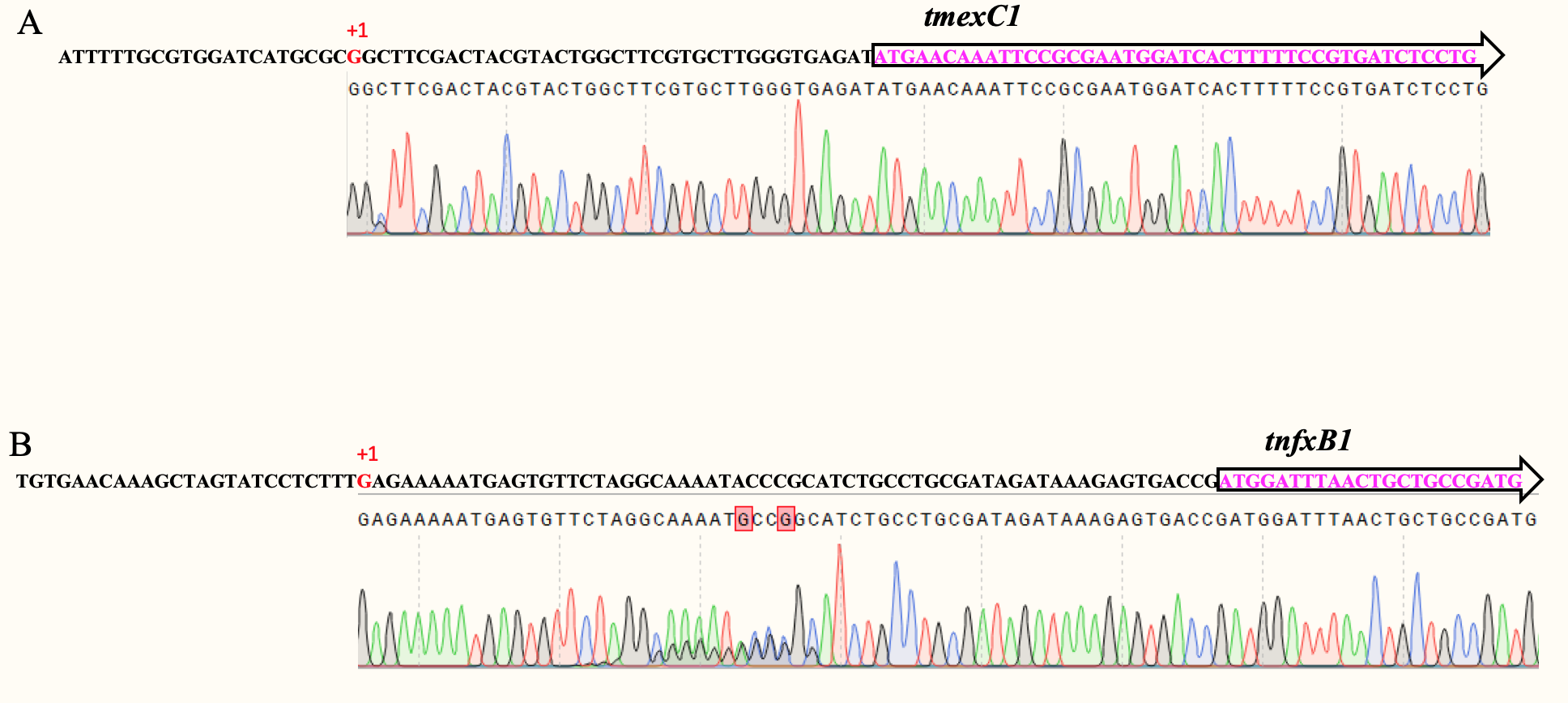


Fig. S11 Sanger sequencing of 5'-rapid amplification of cDNA ends (5'-RACE) results identifying the transcriptional start site of *tmexC1* (A) and *tnfxB1* (B). The part open read frames of TNfxB1 and TMexC1 were boxed.


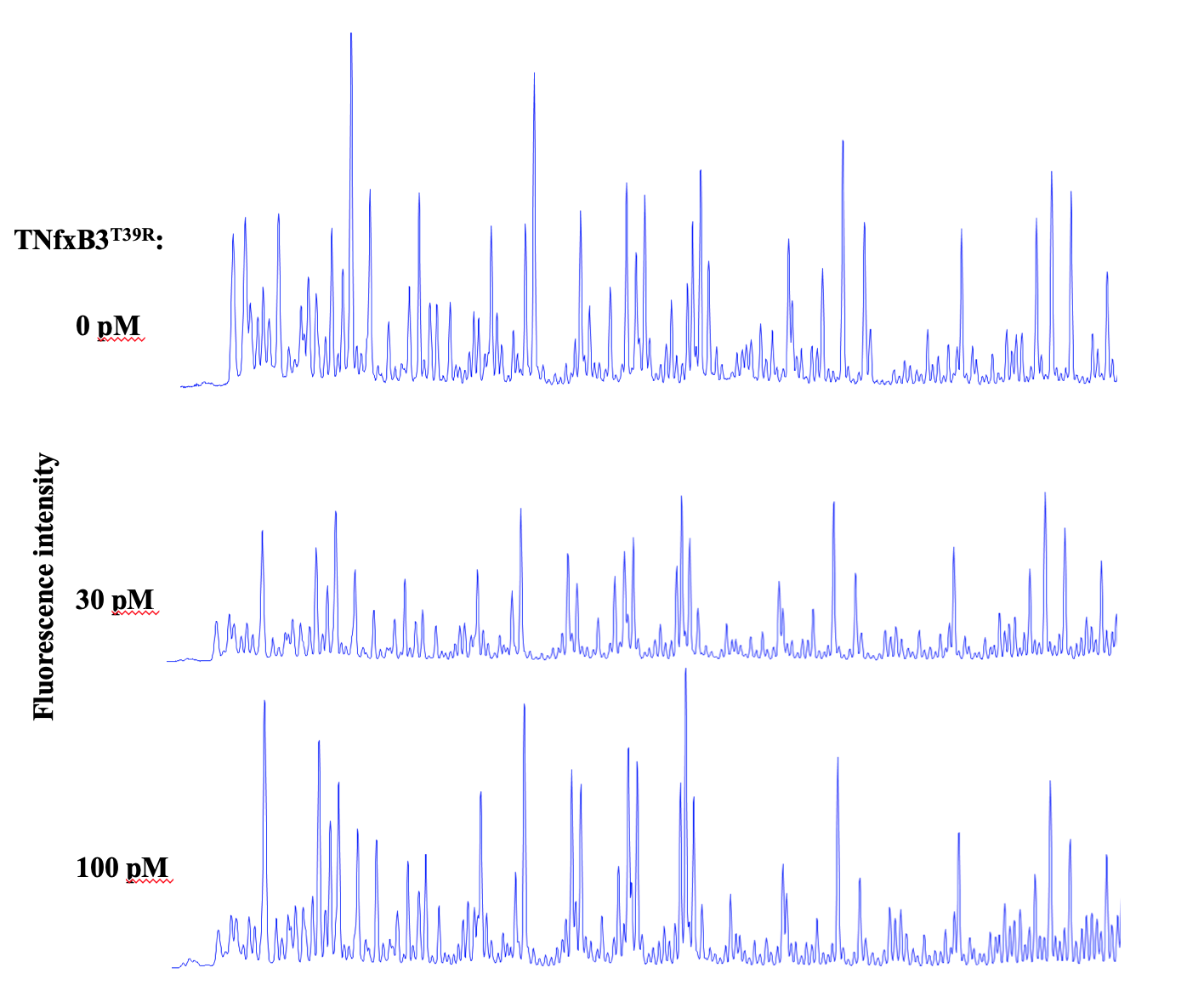


Fig. S12 DNase I footprinting assay showing the binding site of TNfxB3 mutant with arginine 39. The DNA fragment of the FAM-labeled *tnfxB1-tmexC1* intergenic sequence was incubated with TNfxB3^T39R^ protein with two concentration gradients.


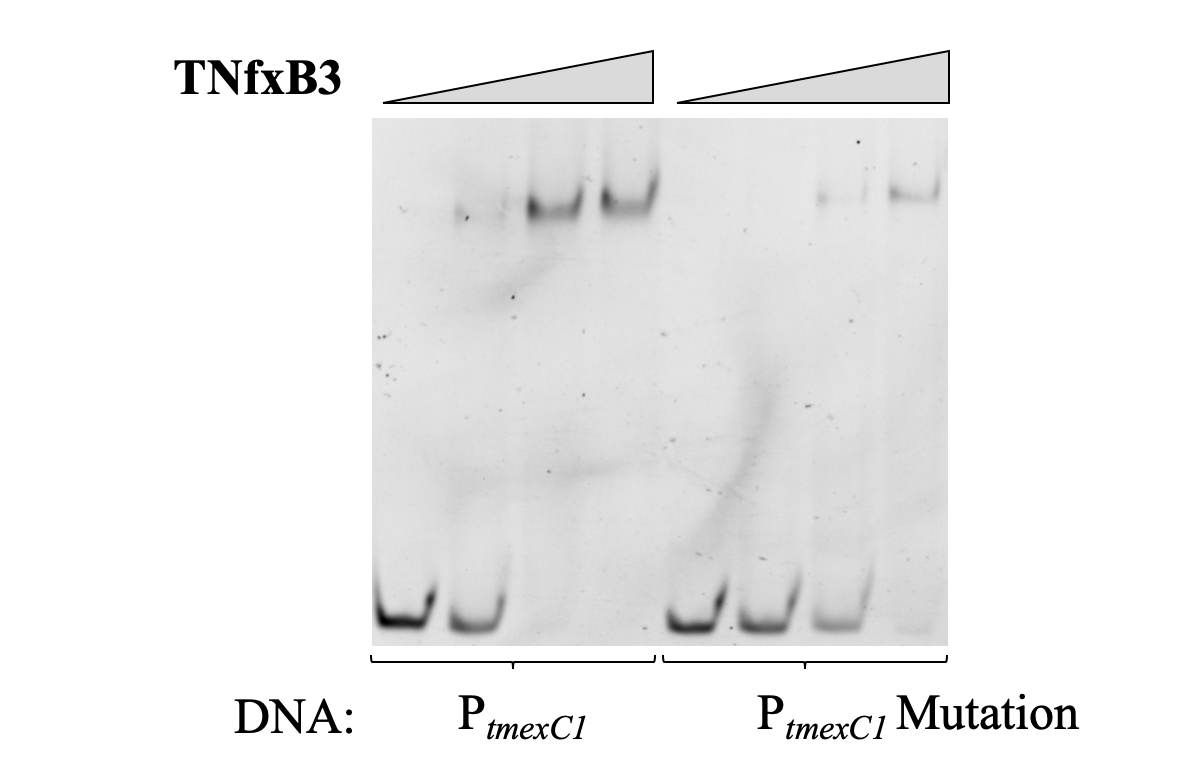


Fig. S13 EMSA analysis of TNfxB3 interaction with operator DNA or its mutant DNA. A mutated DNA fragment was constructed by replacing 28bp A/T into C/G in two inverted repeats shown in Fig. 4C.


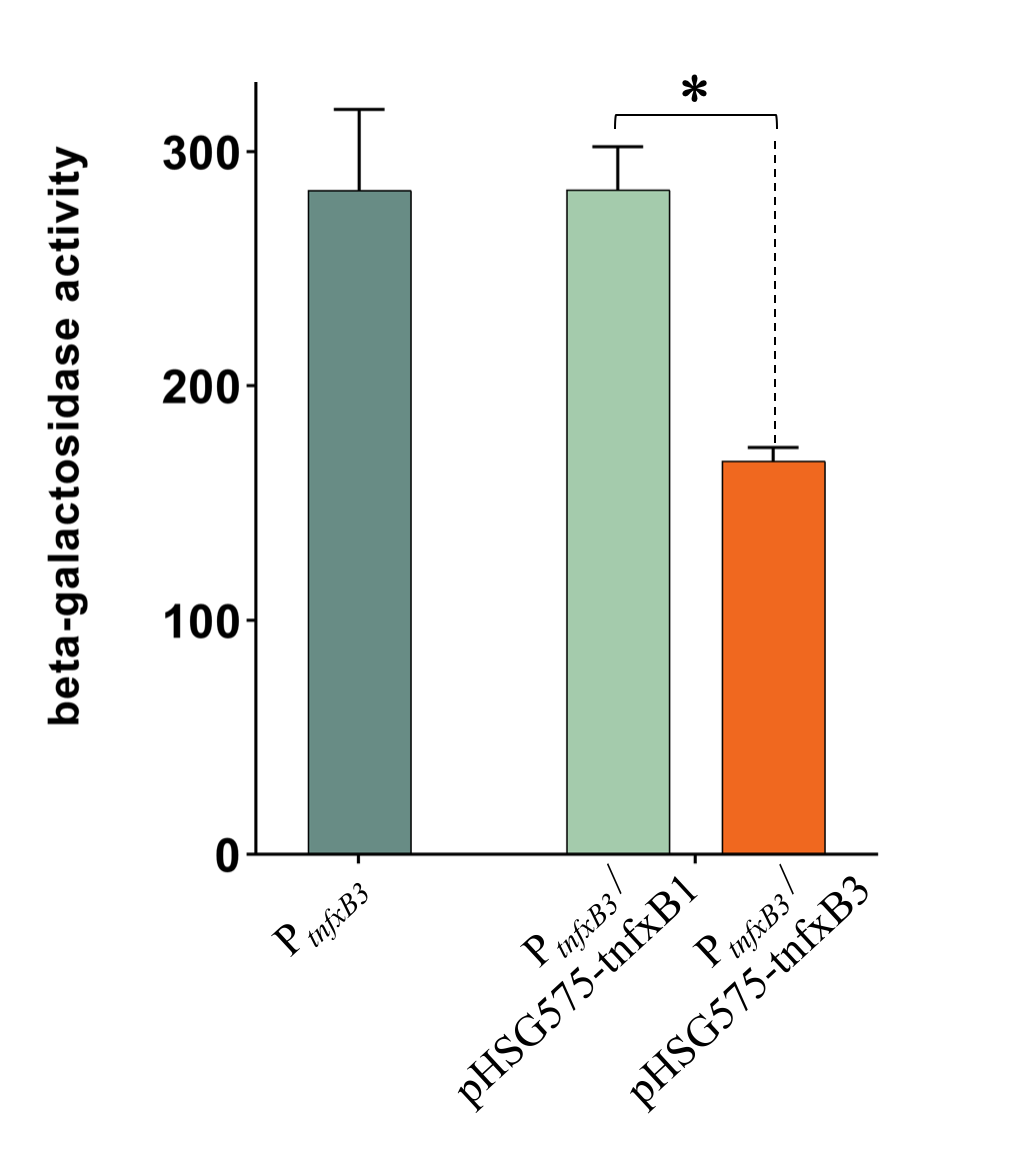


Fig. S14 Auto-repression of regulator TNfxB3 measured by β-galactosidase assay in *vivo*. Statistical analysis was performed through Student's t-tests. *, P < 0.05.


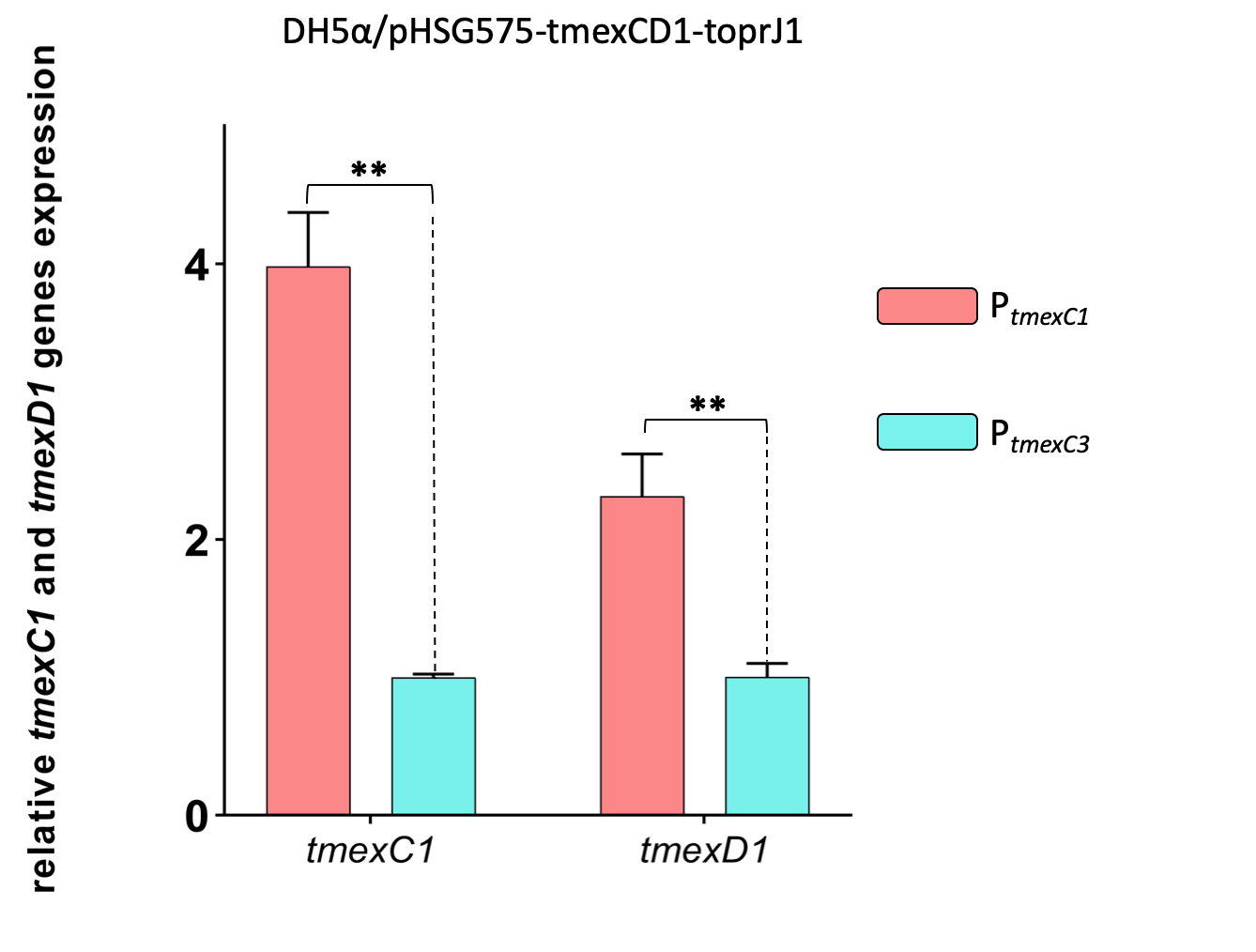


Fig. S15 Relative transcriptional expression level of *tmexC1* and *tmexD1* genes in two recombinant strains carrying *tmexCD1-toprJ1* but with two promoter regions of P*_tmexC1_* or P*_tmexC3_*. Statistical analysis was performed through Student's t-tests. **, P < 0.01.


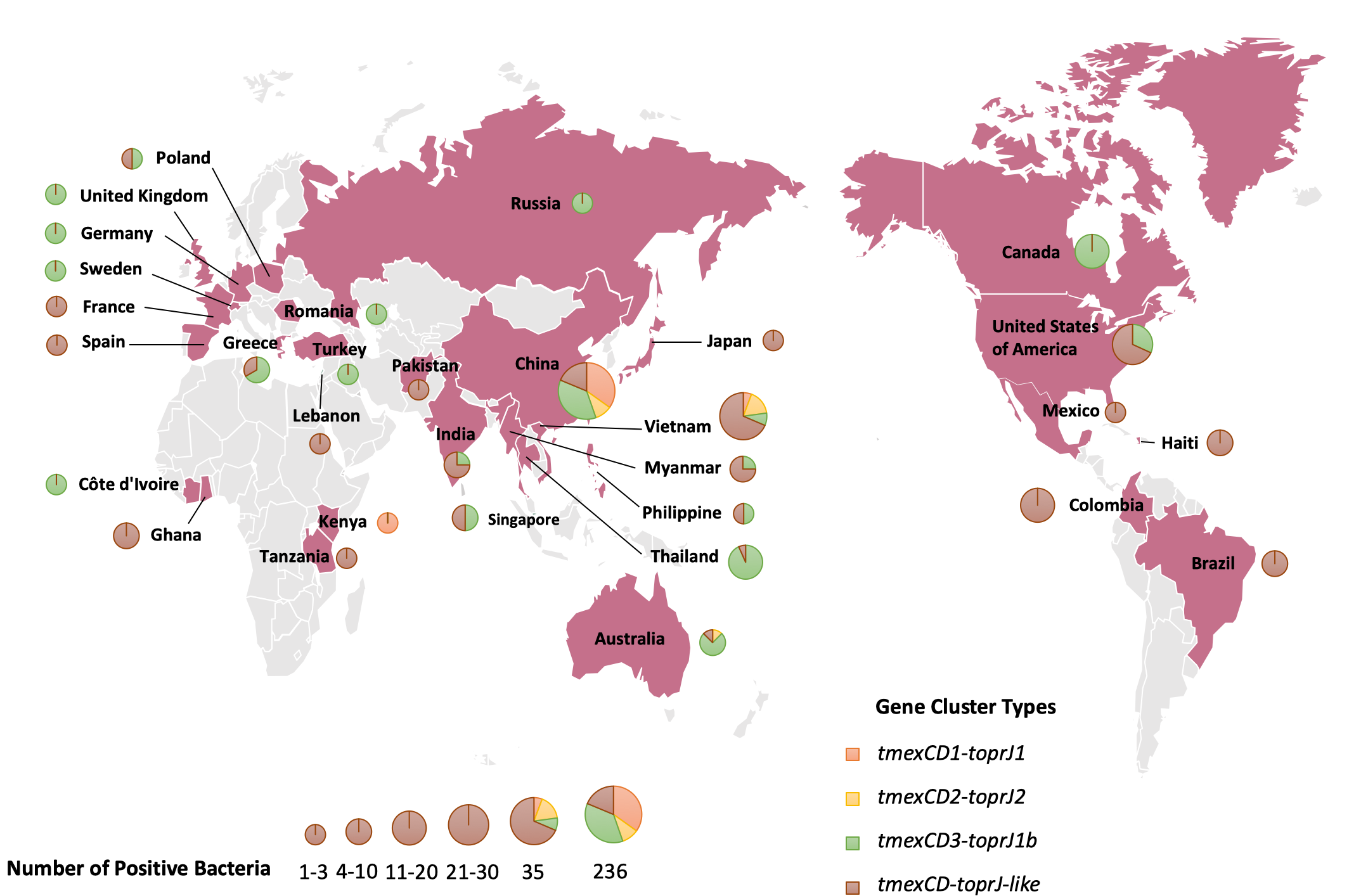


Fig. S16 Global map of *tmexCD-toprJ*-positive bacteria. Data for all 467 strains were obtained from the GenBank database. The 31 countries wherein *tmexCD-toprJ* has been detected are marked with deep red. The four types of *tmexCD-toprJ* are shown in different colors in pie charts. Different sizes of the pie charts represent the numbers of *tmexCD-toprJ*-positive isolates detected in each country and the composition of *tmexCD-toprJ* variants.


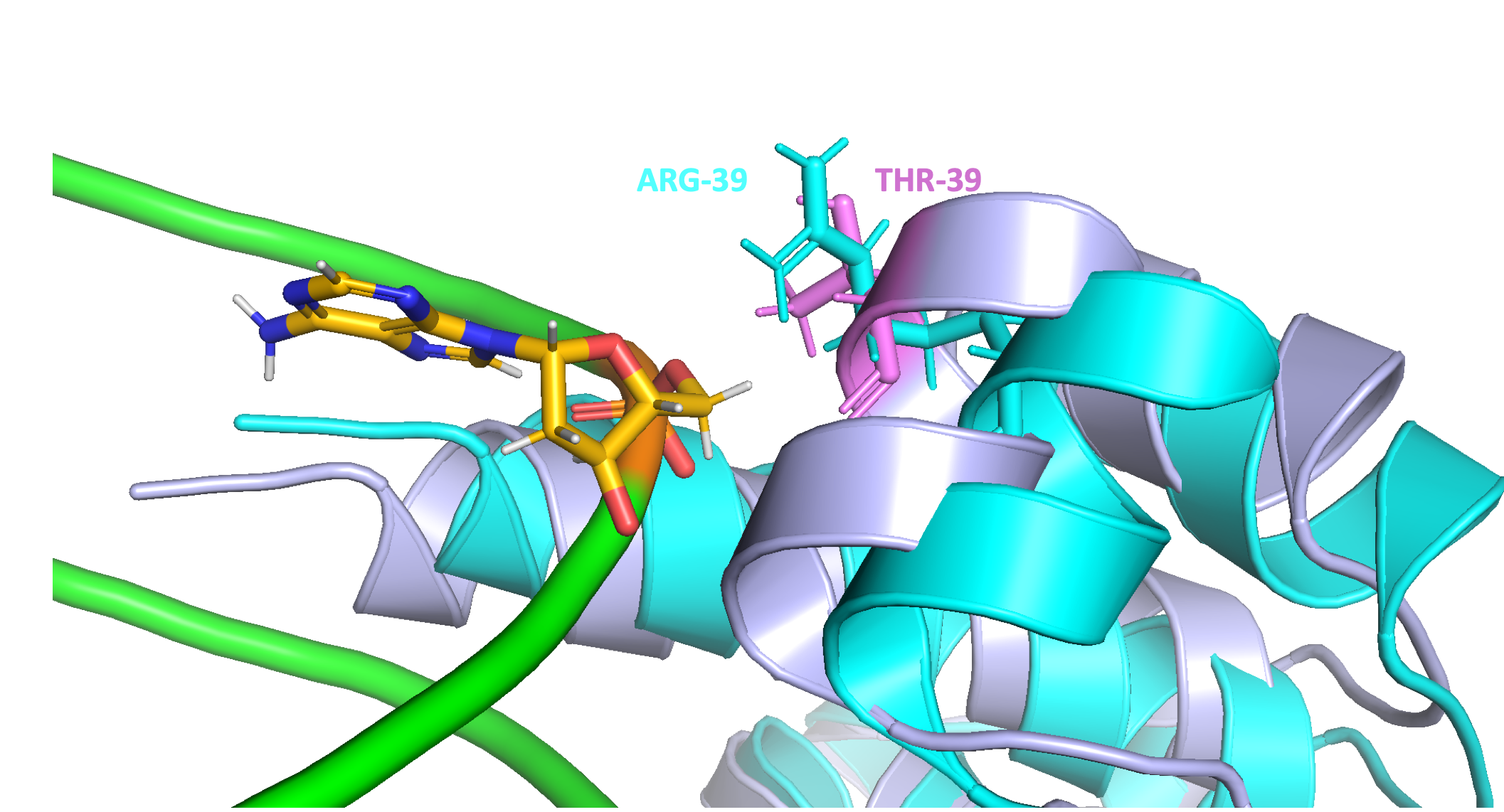


Fig. S17 Comparison of DNA interaction changes after T39R mutation in NfxB by molecular docking. Purple protein represents the wild-type NfxB protein and NfxB^T39R^ mutant protein. Residue 39 is shown as sticks with different colors.

Table S1 Various antibiotics susceptibility profiles (MICs, mg/L) of strains in this study

| Strains | Strain information | TIG | TET | CQM | FEP | STR |
| --- | --- | --- | --- | --- | --- | --- |
| DH5α/pHSG575-tnfxB1-P1-tmexCD1-toprJ1 | transformants expressing *tnfxB1*-*tmexC1D1-toprJ1* operon in promoter of *tmexC1* | 4 | 4 | 1 | 0.5 | 16 |
| DH5α/pHSG575-tnfxB1^R39T^-P1-tmexCD1-toprJ1 | transformants expressing *tnfxB1^R39T^*-*tmexC1D1-toprJ1* operon in promoter of *tmexC1* | 2 | 2 | 0.5 | 0.25 | 8 |
| DH5α/pHSG575-tnfxB1^N56D^-P1-tmexCD1-toprJ1 | transformants expressing *tnfxB1^N56D^*-*tmexC1D1-toprJ1* operon in promoter of *tmexC1* | 4 | 4 | 1 | 0.5 | 16 |
| DH5α/pHSG575-tnfxB1^A72S^-P1-tmexCD1-toprJ1 | transformants expressing *tnfxB1^A72S^*-*tmexC1D1-toprJ1* operon in promoter of *tmexC1* | 4 | 4 | 1 | 0.5 | 16 |
| DH5α/pHSG575-tnfxB1^I88T^-P1-tmexCD1-toprJ1 | transformants expressing *tnfxB1^I88T^*-*tmexC1D1-toprJ1* operon in promoter of *tmexC1* | 4 | 4 | 1 | 0.5 | 16 |
| DH5α/pHSG575-tnfxB3-P1-tmexCD1-toprJ1 | transformants expressing *tnfxB3*-*tmexC1D1-toprJ1* operon in promoter of *tmexC1* | 2 | 2 | 0.5 | 0.25 | 8 |
| DH5α/pHSG575-tnfxB3^T39R^-P1-tmexCD1-toprJ1 | transformants expressing *tnfxB3^T39R^*-*tmexC1D1-toprJ1* operon in promoter of *tmexC1* | 4 | 4 | 1 | 0.5 | 16 |
| DH5α/pHSG575-tnfxB3^D56N^-P1-tmexCD1-toprJ1 | transformants expressing *tnfxB3^D56N^*-*tmexC1D1-toprJ1* operon in promoter of *tmexC1* | 2 | 2 | 0.5 | 0.25 | 8 |
| DH5α/pHSG575-tnfxB3^S72A^-P1-tmexCD1-toprJ1 | transformants expressing *tnfxB3^S72A^*-*tmexC1D1-toprJ1* operon in promoter of *tmexC1* | 2 | 2 | 0.5 | 0.25 | 8 |
| DH5α/pHSG575-tnfxB3^T88I^-P1-tmexCD1-toprJ1 | transformants expressing *tnfxB3^T88I^*-*tmexC1D1-toprJ1* operon in promoter of *tmexC1* | 2 | 2 | 0.5 | 0.25 | 8 |

TIG, tigecycline; TET, tetracycline; CQM, cefquinome; FEP, cefepime; STR, streptomycin.

Table S2 The antibiotics susceptibility profiles (MICs, mg/L) of strains expressing FLAG-tagged *tnfxB* or *nfxB* genes in this study

| Strains | Strain information | TIG | TET | CQM | FEP | STR |
| --- | --- | --- | --- | --- | --- | --- |
| DH5α/pHSG575-P1-  tnfxB1-Flag-tmexCD1-toprJ1 | transformants expressing  *tmexC1D1-toprJ1* together with Flag-tagged *tnfxB1* fusion gene | 4 | 4 | 1 | 0.5 | 16 |
| DH5α/pHSG575-P1-  tnfxB3- Flag-tmexCD1-toprJ1 | transformants expressing  *tmexC1D1-toprJ1* together with Flag-tagged *tnfxB3* fusion gene | 2 | 2 | 0.5 | 0.25 | 8 |
| DH5α/pHSG575-P1-  nfxB-Flag-tmexCD1-toprJ1 | transformants expressing  *tmexC1D1-toprJ1* together with Flag-tagged *nfxB* fusion gene | 1 | 1 | 0.125 | 0.125 | 2 |
| DH5α/pHSG575-P1-  nfxB^T39R^-Flag-tmexCD1-toprJ1 | transformants expressing  *tmexC1D1-toprJ1* together with Flag-tagged *nfxB^T39R^* fusion gene | 4 | 4 | 1 | 0.5 | 16 |

TIG, tigecycline; TET, tetracycline; CQM, cefquinome; FEP, cefepime; STR, streptomycin.

Table S3 Bacterial strains and plasmids used in this study

| Names | Purpose | GenBank  accession number |
| --- | --- | --- |
| *Pseudomonas aeruginosa* PAO1 | To clone *nfxB-mexCD-oprJ* operon | AE004091.2 |
| *Proteus cibarius* SDQ8C180-2T | To clone *tnfxB3-tmexCD3-toprJ1b* operon | CP073356.1 |
| Plasmids: | | |
| pHSG575 | For DNA cloning and recombinant DNA expression | None |
| pHGR | For β-Galactosidase reporter assay | None |
| pMAL-c5x | For protein expression and purification | None |
| pHNAH8I-1 | To clone *tmexCD1-toprJ1* operon | MK347425.1 |
| pHNNC189-2 | To clone *tmexCD2-toprJ2* operon | MN175502.1 |

Table S4 Primers used in this study.

| Primers | Sequence (5'-3') | Purpose |
| --- | --- | --- |
| tnfxB-F | CTGAGCGTTGCTTTCCGTTAAGTTTG | To construct pHSG575-tnfxB-tmexCD-toprJ and pHSG575-nfxB-mexCD-oprJ |
| toprJ-R | GATGCAGGACTCCTGGCTTTGCTC |  |
| RE-p575-R | CCAAACTTAACGGAAAGCAACGCTCAGCGTAATCATGGTCATAGCTGTTTCCTG |  |
| RE-p575-F | GAGCAAAGCCAGGAGTCCTGCATCGAATTCACTGGCCGTCGTTTTAC |  |
| NfxB-F | GTCAGGAGCGAGCCGGATTG |  |
| OprJ-R | GTTTACCCGCTCAACTCCTGCC |  |
| RE-p575-NfxB-R | CGCCTCCAATCCGGCTCGCTCCTGACGCGTAATCATGGTCATAGCTGTTTCC |  |
| RE-p575-NfxB-F | GGCGGCAGGAGTTGAGCGGGTAAACGAATTCACTGGCCGTCGTTTTACAACG |  |
| Inser-tnfxB-F | ATGGATTTAACTGCTGCCGATGAG | To construct pHSG575-tnfxB2-tmexCD1-toprJ1, pHSG575-tnfxB3-tmexCD1-toprJ1, and pHSG575-nfxB-tmexCD1-toprJ1 |
| Inser-tnfxB-R | GGTAGTTGCAAGCCCCGTTCAG |  |
| RE-tnfxB-R | CTCATCGGCAGCAGTTAAATCCAT |  |
| RE-tnfxB-F | CTGAACGGGGCTTGCAACTACC |  |
| Inser-NfxB-F | ATGACCCTGATTTCCCATGACGAG |  |
| Inser-NfxB-R | GTCAGGAGCGAGCCGGATTGGA |  |
| RE-Inser-nfxB-F | CTCGTCATGGGAAATCAGGGTCATCGGTCACTCTTTATCTATCGCGG |  |
| RE-Inser-nfxB-R | GCCTCCAATCCGGCTCGCTCCTGAGCGTAATCATGGTCATAGCTGTTTCC |  |
| RE-toprJ-F | GAGTTGTCCGGATTGTTCGATGG |  |
| RE-toprJ-R | CAGTGCCGAGTTGTCCGGATTG |  |
| RE-p-tnfxB-F | CGGCTTCGACTACGTACTGGCTTCGGAATTCACTGGCCGTCGTTTTAC | To construct pHSG575-tnfxB1 and pHSG575-tnfxB3 |
| pHSG-tnfxB-F | CGAAGCCAGTACGTAGTCGAAG |  |
| pHSG-tnfxB-R | CTGAGCGTTGCTTTCCGTTAAGTTTG |  |
| RE-p-tnfxB-R | CAAACTTAACGGAAAGCAACGCTCAGCCGCTCACAATTCCACACAAC |  |
| Pro-tmexC-F | CTCATCGGCAGCAGTTAAATCCATC | To construct pHSG575-tmexCD-toprJ and pHSG575-mexCD-OprJ |
| RE-Pro-tmexC-R | GATGGATTTAACTGCTGCCGATGAGCGTAATCATGGTCATAGCTGTTTCC |  |
| Pro-MexC-F | GATGAGTCGCTCGTCATGGGAAATC |  |
| RE-Pro-mexC-R | CTGATTTCCCATGACGAGCGACTCATCCGTAATCATGGTCATAGCTGTTTCC |  |
| OprJ-R | TCAACTCCTGCCGCCTCGATGTACC |  |
| RE-Pro-OprJ-R | GGTACATCGAGGCGGCAGGAGTTGA |  |
| 16sRNA-F | TGTAGCGGTGAAATGCGTAGA | To measure relative expression of these genes |
| 16sRNA-R | CACCTGAGCGTCAGTCTTCGT |  |
| qPCR-nfxB-F | CCATGACGAGCGACTCATCAAGG |  |
| qPCR-nfxB-R | GAGCATCTGCACCAGGTTGTC |  |
| qPCR-tnfxB-F | CATTGCCGATGCCGATCTTGAG |  |
| qPCR-tnfxB-R | GAGTAAGGCAGCCATCGGCAG |  |
| qPcr-mexD-F | CTCGAGCTATACGTGCCTAACG |  |
| qPcr-mexD-R | CATGGCTTCGCCGGTACTGAAG |  |
| qPCR-tmexC-F | CTGTTCCAGATCGACCCTGC |  |
| qPCR-tmexC-R | GTTTCGACATTGGCCTGGGC |  |
| qPCR-tmexD-R | CTTCAGCTTCTCCGGCATGG |  |
| qPCR-tmexD-F | CTTGCCCAGCACTTCATCGC |  |
| R1-tmexC-R | GTCGATCTGGAACAGCACGTCAC | To determined the transcription start site with 5′-RACE analysis |
| R2-tmexC-R | GAAGGTGCGCTTGAGCACGATC |  |
| inteG-F | GTGATCCATTCGCGGAATTTGTTCA | To obtain the DNA fragment used in EMSA analysis |
| inteG-R | CTCATCGGCAGCAGTTAAATCCATC |  |
| Dna-inteG-F | CTCATCGGCAGCAGTTAAATCCATC | To obtain the DNA fragment used in DNase I footprinting assay |
| FAM-tmexC-TSS-R | CACCCAAGCACGAAGCCAGTACGTAG |  |
| bio-REP-P1-F | GTCGAAGCCGCGCATGATCC | To obtain the DNA fragment used in SPR analysis |
| bio-M2-F | ATACTAGCTTTGTTCACACCAAAATCAAATGAGTCAATATTGACTTATTTGATTTTTGC |  |
| RE-lacZ-F | GTACTGGCTTCGTGCTTGGGTGAGATATGACCATGATTGATCCGTCGACAAG | To construct recombinant plasmids, pHGR-PtmexC-lacZ, pHGR-PmexC-lacZ, pHGR-tnfxB-PtmexC-lacZ, pHGR-nfxB-PtmexC-lacZ, pHGR-nfxB-PmexC-lacZ |
| RE-lacZ-R-tnfxB | CAAACTTAACGGAAAGCAACGCTCAGCACCGGTTTATTGACTACCGGAAGCAG |  |
| RE-lacZ-R-pro | ATGGATTTAACTGCTGCCGATGAGCACCGGTTTATTGACTACCGGAAGCAG |  |
| tmexC-promoter-F | CATATCTCACCCAAGCACGAAGC |  |
| tmexC-promoter-R | CTCATCGGCAGCAGTTAAATCCATCGG |  |
| tnfxB-R | CTGAGCGTTGCTTTCCGTTAAGTTTG |  |
| nfxB-F | ATGACCCTGATTTCCCATGACGAGC |  |
| nfxB-R | GGTCAGGAGCGAGCCGGATTG |  |
| RE-lacZ-F-NfxB | CCTCCAATCCGGCTCGCTCCTGACCCACCGGTTTATTGACTACCGGAAGCAGTGTG |  |
| RE-lacZ-R-NfxB | CGCTCGTCATGGGAAATCAGGGTCATCGGTCACTCTTTATCTATCGCGGGCAG |  |
| mexC-promoter-F | CTCGTCATGGGAAATCAGGGTCATC |  |
| mexC-promoter-R | CATGACACACCCGACCGTTGA |  |
| RE-lacZ-F-mexC | AATCAACGGTCGGGTGTGTCATGACCATGATTGATCCGTCGAC |  |
| RE-lacZ-R-mexC | GATGACCCTGATTTCCCATGACGAGCACCGGTTTATTGACTACCGGAAG |  |
| PtnfxB-F | GCGGAATTTGTTCATATCTCACCCAAG | To construct recombinant plasmid pHGR-PtnfxB-lacZ |
| PtnfxB-R | CATCGGTCACTCTTTATCTATCGCG |  |
| RE-PtnfxB-pHGR-F | GCCCGCGATAGATAAAGAGTGACCGATGACCATGATTGATCCGTCGACAAG |  |
| RE-PtnfxB-pHGR-R | CTTGGGTGAGATATGAACAAATTCCGCCACCGGTTTATTGACTACCGGAAGC |  |
| PtmexC1-71-F | TTGACTTATTTGATTTTTGCGTGGATCATG | To construct a series of different-sized PtmexC1-lacZ fusion reporters |
| RE-PtmexC1-71-F | TCCACGCAAAAATCAAATAAGTCAACACCGGTTTATTGACTACCGGAAG |  |
| PtmexC1-99-F | GTTCACACCAAAATCAAATGAGTCAATATTG |  |
| RE-PtmexC1-99-F | TGACTCATTTGATTTTGGTGTGAACCACCGGTTTATTGACTACCGGAAG |  |
| PtmexC1-118-F | CAAAGAGGATACTAGCTTTGTTCACACC |  |
| RE-PtmexC1-118-F | GGTGTGAACAAAGCTAGTATCCTCTTTGCACCGGTTTATTGACTACCGGAAG |  |
| PtmexC1-135-F | AGAACACTCATTTTTCTCAAAGAGGATAC |  |
| RE-PtmexC1-135-F | CCTCTTTGAGAAAAATGAGTGTTCTCACCGGTTTATTGACTACCGGAAG |  |
| PtmexC1-M14-L-F | CAATATTGACTTGCCCAGCCCCAACGTGAACAAAGCTAGTATCCTCTTTGAG |  |
| RE-PtmexC1-M14-L-F | CTTTGTTCACGTTGGGGCTGGGCAAGTCAATATTGACTTATTTGATTTTTGCGTGG |  |
| PtmexC1-M14-R-R | GTCAATATTGACTCGCCCAGCCCCCATGTGGATCATGCGCGGCTTCGA |  |
| RE-PtmexC1-M14-R-R | CGCATGATCCACATGGGGGCTGGGCGAGTCAATATTGACTCATTTGATTTTGGTGTG |  |
| check-pHGR01-R | CAACTGGCCTCAGGCATTTGAG |  |
| RE-check-pHGR-R | CTTCTCAAATGCCTGAGGCCAG |  |
| check-pHGR01-F | GCTATTACGCCAGCTGGCGAAAG |  |
| RE-check-pHGR-F | CAGTTGCGCAGCCTGAATGG |  |
| tnfxB-F | ATGGATTTAACTGCTGCCGATGA | To construct pMAL-c5x-tnfxB, pMAL-c5x-nfxB, and their mutants |
| tnfxB-R | AGTTTGGTAGTTGCAAGCCCC |  |
| RE-pMAL-tnfxB-F | TGAACGGGGCTTGCAACTACCAAACTCACCACCACCACCACCACTGAC |  |
| RE-pMAL-tnfxB-R | CTCATCGGCAGCAGTTAAATCCATAATCTATGGTCCTTGTTGGTCAATTGC |  |
| nfxB-F | ATGACCCTGATTTCCCATGACGA |  |
| nfxB-R | GGAGCGAGCCGGATTGGAG |  |
| RE-pMAL-nfxB-F | CGTCATGGGAAATCAGGGTCATAATCTATGGTCCTTGTTGGTCAATTGC |  |
| RE-pMAL-nfxB-R | ATGGCGCCTCCAATCCGGCTCGCTCCCACCACCACCACCACCACTGACC |  |
| FLAG-tnfxB-F | TCAGATCTTATCGTCGTCATCCTTGTAATCAGTTTGGTAGTTGCAAGCCCCG | To construct pHSG575-tnfxB-FLAG-tmexCD1-toprJ1, pHSG575-nfxB-FLAG-tmexCD1-toprJ1, and their mutants |
| FLAG-tnfxB-R | GATTACAAGGATGACGACGATAAGATCTGACGGAAAGCAACGCTCAGCGTAATCATG |  |
| RE-toprJ-F | GAGTTGTCCGGATTGTTCGATGG |  |
| RE-toprJ-R | CAGTGCCGAGTTGTCCGGATTG |  |
| FLAG-NfxB-F | TCAGATCTTATCGTCGTCATCCTTGTAATCGGAGCGAGCCGGATTGGAG |  |
| FLAG-NfxB-R | GATTACAAGGATGACGACGATAAGATCTGACGGAAAGCAACGCTCAGCGTAATCATG |  |
| tnfxB1-R39T-R | GTCAGCAAGGCGACGCTCAACAGGT | To make single point mutation in TNfxB1 or TNfxB3 |
| tnfxB1-R39T-F | ACCTGTTGAGCGTCGCCTTGCTGAC |  |
| tnfxB1-N56D-R | GATCGAGATGCTTCTGGATCATGGTTCG |  |
| tnfxB1-N56D-F | CGAACCATGATCCAGAAGCATCTCGATC |  |
| tnfxB1-A73S-R | GATGCCGATCTTGAGTCGGCGCCCTTGGAC |  |
| tnfxB1-A73S-F | GTCCAAGGGCGCCGACTCAAGATCGGCATC |  |
| tnfxB1-I88T-F | CTCCCTGTGAGTCAAATGGCCTTCGATCAG |  |
| tnfxB1-I88T-R | CTGATCGAAGGCCATTTGACTCACAGGGAG |  |
| RE-R-575-TOprJ1 | GACCTGATAGTTGCTGGTCACTTCCGAG |  |
| RE-F-575-TOprJ1 | CTCGGAAGTGACCAGCAACTATCAGGTC |  |
| tnfxB3-T39R-F | GTCAGCAAGGCGAGGCTAAACAGGT |  |
| tnfxB3-T39R-R | ACCTGTTTAGCCTCGCCTTGCTGAC |  |
| tnfxB3-D56N-F | CGAGATGCTTCTGAATCATGGTTCGGTGG |  |
| tnfxB3-D56N-R | CCACCGAACCATGATTCAGAAGCATCTCG |  |
| tnfxB3-S73A-F | GATCTTGAGGCGGCGCCCTTGGA |  |
| tnfxB3-S73A-R | TCCAAGGGCGCCGCCTCAAGATC |  |
| tnfxB3-F88I-F | CGAAGGCCATTTGATTCACAGGGAGTTG |  |
| tnfxB3-F88I-R | CAACTCCCTGTGAATCAAATGGCCTTCG |  |
| PAO1-nfxB-T39D-R | GGCGTAAGCAAGGCCGACCTGCACCGCTTCTG | To make single point mutation in NfxB |
| PAO1-nfxB-T39D-F | CAGAAGCGGTGCAGGTCGGCCTTGCTTACGCC |  |
| PAO-nfxB-T39R--R | CGTAAGCAAGGCCAGGCTGCACCGCTTCTG |  |
| PAO-nfxB-T39R-F | CAGAAGCGGTGCAGCCTGGCCTTGCTTACG |  |
| PAO-nfxB-T39A-R | CGTAAGCAAGGCCGCCCTGCACCGCTTCTG |  |
| PAO-nfxB-T39A-F | CAGAAGCGGTGCAGGGCGGCCTTGCTTACG |  |
| PAO1-nfxB-T39S-F | GAAGCGGTGCAGGCTGGCCTTGCTTACGC |  |
| PAO1-nfxB-T39S-R | GCGTAAGCAAGGCCAGCCTGCACCGCTTC |  |
| RE-R-pHGR-lacZ | CTCGATGCGATGTTTCGCTTGGTG |  |
| RE-F-pHGR-lacZ | CACCAAGCGAAACATCGCATCGAG |  |
